# Preliminary testing of a prespecified liability architecture for autism: theory-guided pathogenetic triad models outperform strength-matched alternatives

**DOI:** 10.3389/fpsyt.2026.1837909

**Published:** 2026-07-06

**Authors:** Darko Sarovic

**Affiliations:** 1 Institute of Clinical Sciences, University of Gothenburg, Gothenburg, Sweden; 2 Department of Radiology, Harvard Medical School, Massachusetts General Hospital, Boston, MA, United States; 3 Athinoula A. Martinos Center for Biomedical Imaging, Department of Radiology, Massachusetts General Hospital, Charlestown, MA, United States; 4 Paediatric Health Professions and Paediatric Radiology, Sahlgrenska University Hospital, Gothenburg, Sweden; 5 Department of Women’s and Children’s Health, Uppsala University, Uppsala, Sweden

**Keywords:** autism, autistic traits, heart rate variability (HRV), liability architecture, multilevel framework, multiverse analyses, predictive modeling, pathogenetic triad

## Abstract

**Background:**

Neuropsychiatric conditions are heterogeneous and mechanistically diverse, yet predictive modeling studies commonly aggregate features without evaluating prespecified liability architectures. The Pathogenetic Triad (PT) is a multilevel framework proposing that diagnostic outcomes reflect the joint configuration of a trait-related domain, cognitive capacity (CC), and neuropathological burden (NB). In autism, the trait-related domain corresponds to autistic personality (AP). We evaluated this framework using out-of-sample prediction as a structured empirical test of architectural coherence rather than as a purely data-driven exercise in classification.

**Methods:**

We analyzed a case–comparison cohort (*n* = 42, 21 autistic) with dense multimodal characterization, including behavioral phenotyping, psychometric measures, autonomic physiology, structural MRI morphometry, and magnetoencephalographic indices. AP was indexed by the Autism-Spectrum Quotient, CC by Wechsler’s scales of intelligence, and NB by heart-rate variability as a proxy indicator. This multimodal structure enabled direct comparison of theory-constrained PT models with strength-matched, domain-restricted atheoretical combinations. Predictive discrimination was evaluated using leakage-free nested cross-validation with permutation inference.

**Results:**

Across multiverse specifications, low-dimensional PT models ranked among the strongest models of comparable size and achieved discrimination broadly comparable to higher-dimensional models within this dataset. Matched comparisons indicated that including all three PT domains was associated with systematic advantages relative to alternatives with similar univariate input strength, consistent with complementary configurational information relating to case status.

**Conclusions:**

These findings provide preliminary evidence supporting the Pathogenetic Triad as a multilevel architecture of autism liability. Although based on a small and demographically restricted cohort, this densely characterized dataset permitted explicit comparison of theory-guided and atheoretical model spaces. The results illustrate how prespecified multilevel frameworks can be operationalized and empirically evaluated in neuropsychiatric samples.

## Introduction

Autism spectrum disorder is a neurodevelopmental condition characterized by differences in social communication, restricted and repetitive behaviors, and atypical sensory processing (3). However, autistic presentations are highly heterogeneous, varying in symptom profile, cognitive ability, adaptive functioning, and co-occurring psychiatric and medical conditions (4, 5). This heterogeneity complicates formulation of mechanistic models that capture commonality while respecting phenotypic diversity.

In response to this heterogeneity, biomarker discovery and multivariable classification efforts have accelerated (6, 7). Yet many models are assembled in a largely data-driven way without an explicit mechanistic framework constraining feature selection or structure. This can yield high-dimensional models that are difficult to interpret and generalize, and hard to translate into testable hypotheses (8–10). A theory-guided feature space can impose structure, improve parsimony and interpretability, and link predictive performance to liability architecture, potentially supporting clinical uptake.

These limitations reflect two gaps. First, most mechanistic accounts of autism focus on a single level of analysis and rarely specify how levels should be linked, limiting their value as global explanations. Second, even when multilevel theories are proposed, they are rarely operationalized into specific variables and quantitative predictions suitable for multivariable models (11, 12). As a result, the field lacks a widely adopted multilevel framework that is directly testable in predictive evaluation pipelines.

A related issue concerns which features enter predictive models. Markers that are relatively homogenous across autistic individuals may yield small but stable effects, whereas mechanisms prominent in only subsets may produce larger effects but generalize poorly. Without a principled account of autism’s latent structure, it is unclear how to combine these signals in a way that is both mechanistically meaningful and statistically robust. More generally, even sophisticated algorithms can only operate within the feature space they are given–so defining that space is itself a theoretical act. We refer to this as theory-guided predictive testing: using a multilevel framework to map observed variables onto a small set of interpretable latent dimensions and then evaluating whether this prespecified architecture carries non-redundant information aligned with case-status. The goal is not to optimize a classifier but to test whether the proposed liability structure survives empirical testing.

The Pathogenetic Triad (PT) is a multilevel framework for neurodevelopmental liability (13, 14) linking genetic, biological, cognitive and behavioral measures through measurable components and mechanisms. Although formulated as a neurodevelopmental liability framework, the present study evaluates the PT using autism as a specific and well-characterized test case. The model proposes that autistic outcomes reflect the configuration of three interacting dimensions: Autistic Personality (AP), Cognitive Capacity (CC), and Neuropathological Burden (NB). AP indexes autistic traits; CC captures cognitive resources that can amplify or contain the behavioral expression of AP; and NB reflects neurobiological vulnerability that may constrain compensatory capacity. Within the PT framework, NB refers to burden-related developmental constraint rather than to any single downstream biomarker. Individual biomarkers may reflect aspects or consequences of such constraint, but the construct is not represented by any single marker. In the present pilot test, NB was operationalized using short-term vagally mediated heart-rate variability (HRV), as a pragmatic proxy indicator rather than a direct measure of neuropathology, because autonomic flexibility is theorized to modulate how biological and psychosocial stressors are buffered and translated into developmental constraint, thereby limiting compensatory capacity over time (15, 16). The framework yields testable postulates, including that autistic and non-autistic individuals occupy different regions in triad space and that joint triad-based models should capture configurational information beyond isolated indicators (assessed through the first two questions below).

To our knowledge, PT-guided models have not been tested within formal predictive evaluation pipelines, and few multilevel theories have been evaluated as explicit, operationalized feature spaces for multivariable prediction. If AP, CC, and NB approximate latent dimensions organizing autistic and non-autistic individuals, then a small set of triad-based indicators should capture much of the diagnostic signal; alternative operationalizations should perform similarly; and high-dimensional models should add only modest incremental value beyond the triad. Observing this pattern would support the PT as a plausible liability framework. We treat predictive modeling as a test of mechanistic structure rather than pattern recognition. Accordingly, we emphasize relative model ranking, stability across multiverse specifications, and robustness under strict leakage control, rather than interpreting performance estimates as stable population parameters. We evaluate this logic in a case–comparison cohort using four interrelated questions.

**Does a prespecified PT operationalization show out-of-sample separation?** Specifically, does a simple, low-dimensional PT representation capture discriminative structure aligned with case status under conservative, leakage-free evaluation?

**Do theory-constrained triad-models outperform data-driven alternatives?** If the framework approximates core liability dimensions, PT-consistent models should rank among the highest-performing models of similar size relative to atheoretical combinations matched on univariate input strength.

**How parsimonious is the PT representation of autism risk?** To what extent can a low-dimensional, theory-constrained representation recover the discriminative signal observed in higher-dimensional, atheoretical benchmarks?

**Can structured mapping of PT-consistent operationalizations provide a blueprint for preregistered cumulative testing across samples?**

Together, these analyses evaluate the PT as an empirically useful multilevel framework and illustrate a practical approach to theory-guided predictive testing.

## Materials and methods

### Participants and design

We analyzed an existing IQ-matched case–comparison cohort of autistic and non-autistic men that has been described elsewhere (17, 18). Autistic participants were recruited from existing longitudinal studies where they had received a diagnosis of autism spectrum disorder on at least two separate occasions, using full diagnostic neuropsychiatric multidisciplinary evaluations, including the Autism Diagnostic Observation Schedule. Individuals with other neuropsychiatric diagnoses, such as Attention-Deficit/Hyperactivity Disorder, were excluded. Non-autistic participants were recruited from Gothenburg, Sweden using flyers and on university campus, and individuals with any self-reported psychiatric diagnosis were excluded. The groups were matched for full-scale IQ (FSIQ) and participants with a FSIQ below 80 were excluded.

Analyses used complete-case subsets defined by the predictors required for each model family. The full cohort (*n* = 42; 21 autistic) was used for the PT-complete analyses (primary PT model and TriadIndex). Analyses requiring the expanded predictor set used the largest complete-case subset across those variables (*n* = 35; 17 autistic). The study was approved by the regional ethical board in Gothenburg, Sweden (DNR: 552-14) and all participants provided written informed consent.

### Measures

#### PT operationalization and study-specific operationalization

The PT is a theoretical framework defined at the level of latent domains. The present study does not claim to measure those domains exhaustively. Rather, it tests a pre-specified low-dimensional operationalization in which AQ, WMIQ, and HRV-derived metrics serve as indicator-level proxies for AP, CC, and NB within this dataset.

AP was indexed using the Autism-Spectrum Quotient total score (AQ) (19). It was used here as a pragmatic, continuous trait-level proxy for AP, but AP as formulated in the PT is broader than any single questionnaire score. AQ total may underrepresent aspects of AP, and some items may capture variance related to cognition or internalizing symptoms rather than AP per se.

CC was assessed with the working memory subscale (WMIQ) of Wechsler Adult Intelligence Scale (WAIS-IV) (20). WMIQ is a plausible partial proxy for compensatory capacity, but compensation is multi-faceted and unlikely to be exhausted by a single instrument. Broader executive, language-mediated, adaptive, and social-cognitive resources also contribute to compensation. Additional IQ indices were evaluated in exploratory multiverse analyses.

NB was operationalized using cardiac HRV derived from ECG, with lower vagal modulation interpreted as higher putative burden (21). The primary NB measure was SD1, a Poincaré-plot short-term vagally mediated HRV metric computed from interbeat intervals. HRV is used here as a theory-guided proxy for burden-related developmental constraint, not as a direct measure of neuropathology. Resting vagal HRV is mechanistically plausible because autonomic flexibility may index how biological and psychosocial insults are buffered and translated into developmental constraint, but it is state-sensitive and captures NB only partially. ECG preprocessing and artifact handling are described in the Supplement. Additional HRV metrics were evaluated in exploratory multiverse analyses.

For the primary 2D representation, CC and NB were combined into a theory-guided composite vulnerability axis because, within the PT framework, NB is hypothesized to constrain compensatory capacity, and the two variables therefore jointly define a nonspecific compensation-constraint dimension that modulates whether AP translates into diagnosable impairment. This parsimonious 2D projection facilitates visualization and low-dimensional testing, but should not be interpreted as evidence that CC and NB form a single empirically derived latent factor; accordingly, we also evaluated the 3D PT model with CC and NB modeled separately.

#### Non-PT predictors

To position PT-based models within a broader, data-driven “model space”, we included candidate predictors from three additional domains:

Behavioral (BEH): available self-report measures of alexithymia (Toronto Alexithymia Scale-20) (22) and sensory processing (Adult/Adolescent Sensory Profile total score and subscales) (23).

Anatomical (ANAT): selected structural magnetic resonance imaging-derived (MRI) morphometry measures, including hippocampus and amygdala volumes and global grey- and white-matter volumes.

Neurophysiological (MEG): discriminative magnetoencephalographic (MEG) markers of visual face processing (M130/M170 amplitude and latency, and a late component amplitude).

Within the PT framework, anatomical and functional neurobiological variables are considered developmental end-result biomarkers and were therefore analyzed as separate neurobiological benchmark domains rather than treated as interchangeable indicators of NB. MRI and MEG preprocessing pipelines are presented in (17, 18). Complete lists of included variables and their domain assignments are provided in the Supplement.

### Data processing and univariate characterization

Continuous variables were screened for right-skewed non-normality (Shapiro–Wilk *p* < 0.05; skewness > 0.5) and log-transformed when indicated. Predictors were then oriented so higher values reflected higher autism liability (if univariate area under ROC (AUC) < 0.5), values were sign-flipped) and then z-scored for descriptive summaries (for supervised models, z-scoring was performed within each outer training set). Univariate strength was summarized as AUC = max(AUC, 1–AUC), and model “input strength” was defined as the mean AUC of included predictors.

### Analytic overview

Analyses addressed the four prespecified questions described in the Introduction. Except for the high-dimensional landscape, models were evaluated using leakage-free nested leave-one-out cross-validation (LOOCV) (24): each participant served once as the held-out test case, and all preprocessing, feature construction, and model fitting were confined to the corresponding outer training set. Predictive evaluation used ridge-regularized logistic regression; predictors were z-scored within each outer training set and applied to the held-out participant. The ridge penalty (C) was tuned within each outer training set via inner stratified cross-validation optimizing log loss. Performance was estimated from out-of-fold predictions using AUC (discrimination), balanced accuracy (thresholded performance), and the Brier score (calibration). Uncertainty was quantified using bootstrap confidence intervals over paired out-of-fold predictions and labels, and the primary model additionally used label-permutation testing with the full pipeline refit per permutation. Unless noted, comparisons within a model family used complete-case subsets to avoid differences in missingness. No prospective power calculation was performed because this convenience pilot was designed for leakage-controlled out-of-sample estimation rather than detection of small effects. Analyses were performed in Python 3.11; dependencies and a reproducibility guide (including scripts and synthetic data (1)) are provided in the Supplement.

### PT model performance

We evaluated a prespecified PT operationalization comprising AQ as AP, WMIQ as CC, and SD1 as NB. Consistent with the PT theory, we operationalized the primary two-dimensional PT decision space using standardized AP and an NB/CC composite axis combining neuropathological liability and cognitive capacity, using the mean of their z-scores (z(CC) + z(NB))/2.

Permutation and null performance. Statistical significance of the model’s discrimination was assessed using label-permutation testing, repeating the full nested CV pipeline across 20,000 permutations to obtain a null distribution of AUC and an empirical *p*-value.

Unsupervised structure check. As a descriptive check of structure in PT space, we applied K-means clustering (*K* = 2) to the full-sample coordinates (z(AP), z(NB/CC)). Cluster labels were aligned to diagnosis to maximize agreement, and the resulting operating point (sensitivity/specificity) was reported descriptively.

### The Triadindex as a continuous PT risk score

We examined whether the triad’s information could be collapsed onto a single continuous theory-guided risk axis. We constructed a TriadIndex (TI) by computing the weighted sum of the same three PT variables (standardized and risk-oriented) into a single index (re-standardized across the raw TI) intended to reflect overall PT-consistent liability. The primary TI used a theory-guided weighting (emphasizing AP with a ratio of 2:1:1); an equal-weight TI was evaluated as a theory-agnostic sensitivity variant. TI analyses used complete cases with data for the PT variables (*n* = 42).

### Constructing the triplet multiverse space

We constructed a multiverse of all triplet (*k* = 3) ridge logistic regression models under a domain restriction: each triplet contained three predictors drawn from three distinct domains (no repeated domains within a triplet) from the six available domains (AP, CC, NB, BEH, ANAT, MEG), preventing models from being dominated by a single measurement domain. To ensure fair comparisons, we included individuals with complete data across the full predictor set (*n* = 35). We focused on k = 3 because triplets are the model size that represents the full PT configuration while remaining comparable to data-driven triplets of the same size.

Each model was annotated by its mean univariate AUC (input strength), and by the number of PT-domains it contains: “PT triads” were defined as triplets containing all three PT domains (3PT); non-PT triads were defined by the number of PT domains as 0PT, 1PT and 2PT.

#### Matched paired comparisons of PT vs non-PT triplets

To test whether PT triplets outperform data-driven alternatives after controlling for univariate signal, we compared each 3PT triplet to matched triplets from the 0PT pool and an AP-only 1PT pool. For each 3PT triplet, comparator triplets were selected by nearest-neighbor matching on model “input strength” (mean univariate AUC); comparator performance was summarized as the mean AUC across the K matched models (primary K = 5). Sensitivity analyses used K = 1 and profile-based matching on the three predictor-level AUC values with K = 5 (see **Supplementary Methods**). For each 3PT triplet, we computed $\Delta \text{AUC}={\text{AUC}}_{3\text{PT}}-{\text{AUC}}_{\text{matched}}$ and tested for a systematic advantage ( ΔAUC>0) across triplets using Wilcoxon signed-rank tests. Analyses were run for 3PT vs 0PT and for 3PT vs AP-only 1PT (to isolate added value beyond AQ) and summarized in matched-effect plots.

#### Exploratory identification of candidate PT triplets

Within the same k = 3 multiverse results, we identified all PT triads and ranked them by AUC. We also computed the median AUC across all PT triads in which that predictor appeared to indicate which one contained the most discriminative signal.

### Comparing a parsimonious PT-model with a high-dimensional benchmark

We contrasted parsimonious PT-based models (our primary model as *k* = 2 and *k* = 3; 2D; 3D) against a high-dimensional ridge logistic regression benchmark (*k* = 28; 28D) including the full available predictor set in the expanded domain space.

### High-dimensional landscape

To characterize how performance scales with model size in the expanded predictor space, we generated a high-dimensional model landscape by evaluating models of size *k* = 1 to *k* = 28 derived from 2,000 random orderings of the full multivariable predictor set. For each random ordering, we formed nested models (first 1 predictor, first 2 predictors, …, all predictors) and evaluated each model under LOOCV using a fixed ridge penalty *C* selected from the tuned 28D high-dimensional benchmark model to avoid embedding a full inner-loop tuning within the landscape computation. This produces a leakage-free description of the typical relationship between dimensionality and cross-validated discrimination in the expanded predictor space while keeping the landscape computationally tractable.

As an additional check focused on direct fairness for low dimensional models, we also evaluated all *k* = 2–3 models (exhaustive enumeration of all domain-restricted predictor combinations; *n* = 318 and 1856 respectively), enabling direct comparison between the PT models and theory-agnostic low-dimensional subsets from the expanded predictor space.

### Generative AI assistance

A large language model (ChatGPT 5.2: OpenAI) was used to assist with language editing, clarity of exposition, code refactoring, and identifying formatting errors. It was not used to generate results, perform statistical analyses or interpret findings. No model outputs were accepted without author verification.

## Results

### Triad variables and univariate diagnostic resolution

The diagnostic resolution of all candidate predictors and the group distributions for the three core PT components in the primary model are presented in **Supplementary Figure 1** and **Supplementary Table 2**. Across the full predictor set, univariate in-sample AUC ranged from 0.526 to 0.882.

For the prespecified primary model, there were group differences between all three predictors (AQ Total: autism = 24.6 ± 8.9, non-autism 11.9 ± 6.0, *p* = 3×10^-6^, whole sample = 18.2 ± 9.7; WMIQ: autism = 103.3 ± 13.9, non-autism 114.0 ± 12.4, *p* = 0.01, whole sample = 108.6 ± 13.9; SD1: autism = 124.3 ± 63.1, non-autism 185.1 ± 53.9, *p* = 0.002, whole sample = 154.7 ± 64.8). As expected given FSIQ matching, the groups did not differ meaningfully in FSIQ (*p* = 0.27). Because AQ was the strongest single predictor, we also evaluated an AQ-only comparator model that, under the same leakage-free nested pipeline, yielded AUC = 0.862, Brier = 0.163, and BA = 0.833 (**Supplementary Table 3**).

### Does a prespecified PT operationalization show out-of-sample separation?

#### Primary PT model in triad space

**Figure 1:**
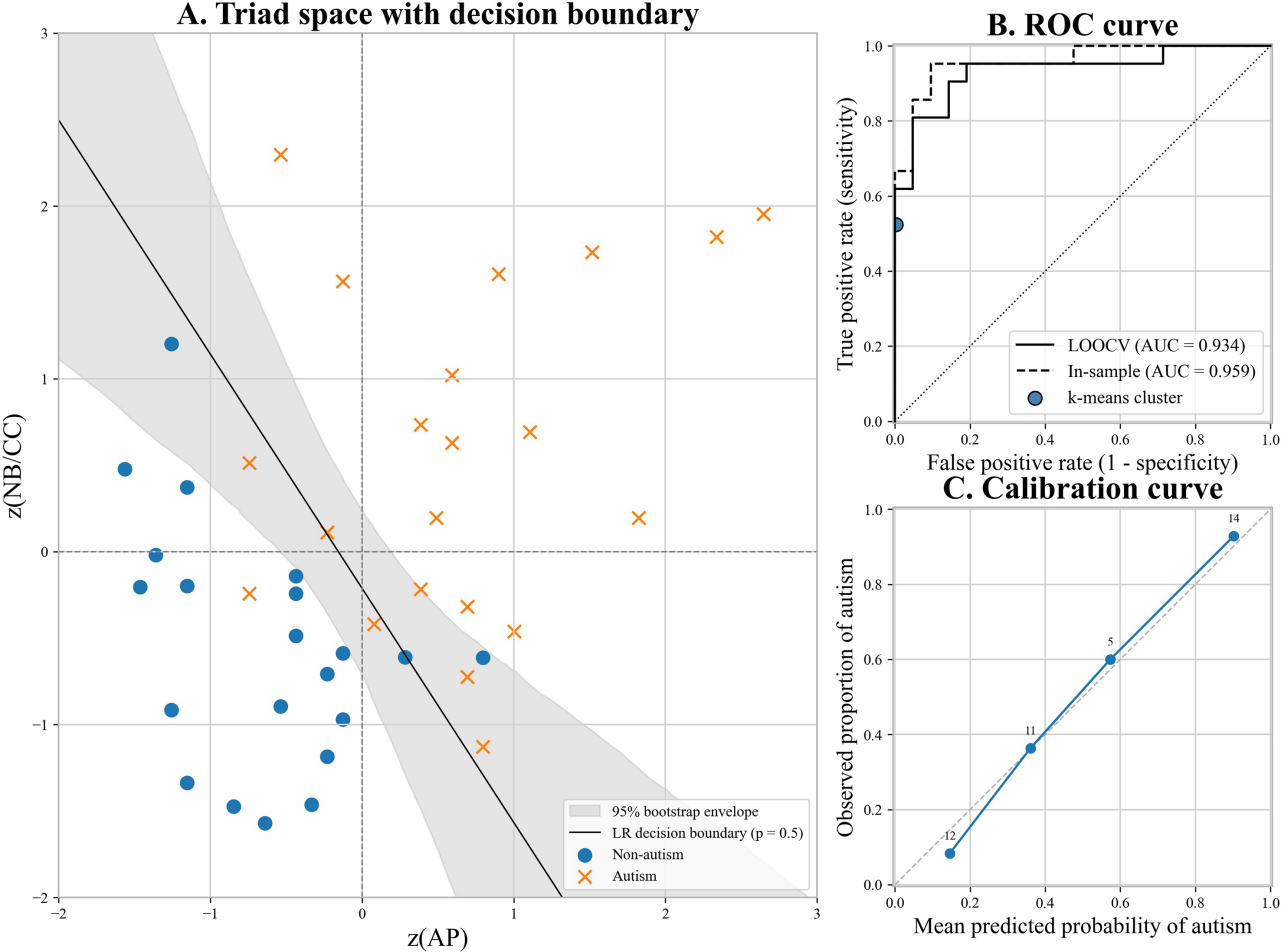
Primary PT model in triad space: decision boundary, ROC and calibration. **(A)** Triad space showing the p = 0.5 decision boundary (shaded 95% bootstrap envelope). **(B)** ROC curves, highlighting the operating point of the unsupervised k-means clustering solution (blue marker). **(C)** Calibration plot (number indicates bin size). LR, logistic regression; AP, autistic personality; NB/CC, composite neuropathological burden/cognitive capacity axis; CI, confidence interval; ROC, receiver operating characteristic; AUC, area under the ROC curve.

In two-predictor triad space, autistic and non-autistic participants occupied largely distinct regions, with an expected diagonal decision boundary (**Figure 1**).

Discrimination was high: AUC = 0.934 (95% CI 0.845–0.995). The BA-maximizing threshold yielded BA = 0.881 (95% CI 0.813–0.978), with sensitivity = 0.952 and specificity = 0.810. The Brier score was 0.114 (95% CI 0.069-0.165).

#### Permutation test and unsupervised clustering

The label-permutation test confirmed that observed performance is highly unlikely under the null. The observed AUC lay far above the 95^th^ percentile of the null distribution (empirical *p* = 5×10^-5^) (**Supplementary Figure 2**).

As a complementary unsupervised check, k-means clustering showed above-chance alignment with case status (BA = 0.762, sensitivity = 0.524, specificity = 1.000). The clustering operating point lay on the ROC curve of the supervised PT model (**Supplementary Figure 2**), consistent with structure in the triad space aligned with case status in this cohort, even without access to labels.

#### TriadIndex: collapsing triad space onto a single axis

The TI model achieved AUC = 0.943 (95% CI 0.857–1.000) and BA = 0.893 (95% CI 0.767–1.000) with Brier = 0.108 (95% CI 0.063–0.163). An equal-weight TI sensitivity model performed similarly (AUC = 0.948 [0.872–0.995]). The results (triad index with decision boundary, ROC curve and calibration curve) are presented in **Supplementary Figures 3**, **4**.

### Do theory-constrained PT models outperform data-driven models with comparable univariate signal?

#### PT triplets in the broader multiverse of triplet models

We next evaluated the multiverse of domain-restricted triplet (*k* = 3) ridge models, comprising a total of 1856 triplets. Model performance increased strongly with predictor input strength (Spearman’s *ρ* = 0.86).

**Figure 2:**
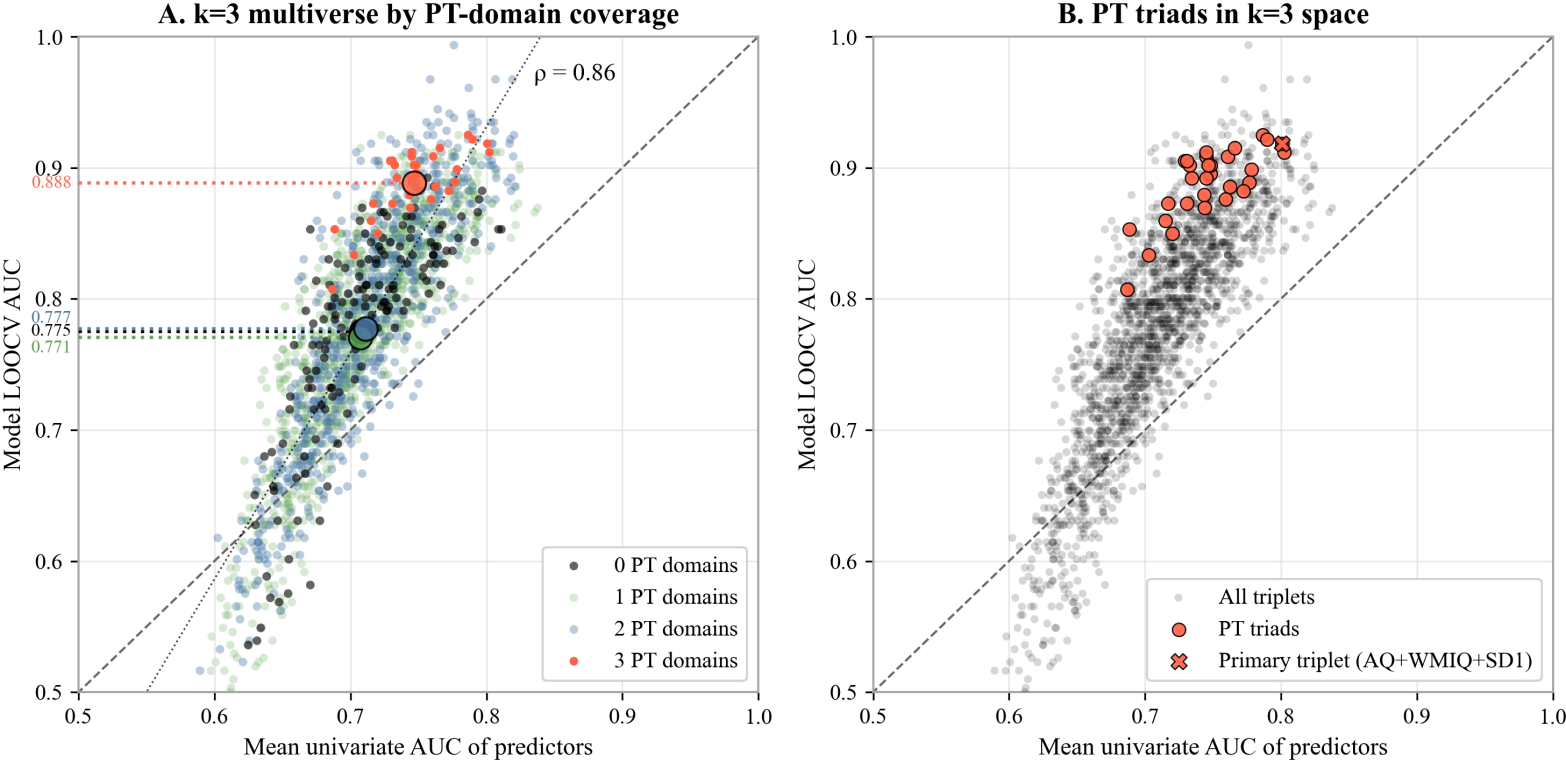
PT-consistent triplets within the triplet multiverse and the link between predictor “input strength” and multivariable performance. **(A)** Model AUC vs mean univariate AUC across triplet multiverse, colored by PT-domain count. Large markers indicate mean AUC within each PT-domain-coverage group. **(B)** Same space highlighting PT-consistent triads and the primary PT triplet. PT-domain count, number of PT domains represented (AP/CC/NB); k, number of predictors.

Models stratified by PT-domain content included 150 0PT triplets, 1020 1PT, 656 2PT, and 30 3PT triplets. Within this space, PT triplets tended to cluster among the highest-performing models at a given level of input strength (**Figure 2**). The pattern of high-performing models across triplet predictors indicates that the triad structure is not tied to one idiosyncratic choice of CC or NB proxy: several different operationalizations of CC and NB work well, as long as all three domains are represented. A supplementary restricted-domain analysis further showed that, among triplets of the form AP + CC + X, models with NB as the third domain tended to occupy a higher performing region than analogous substitutions from BEH, ANAT, or MEG (**Supplementary Figure 6**).

#### Paired comparisons: PT vs matched non-PT models

**Figure 3:**
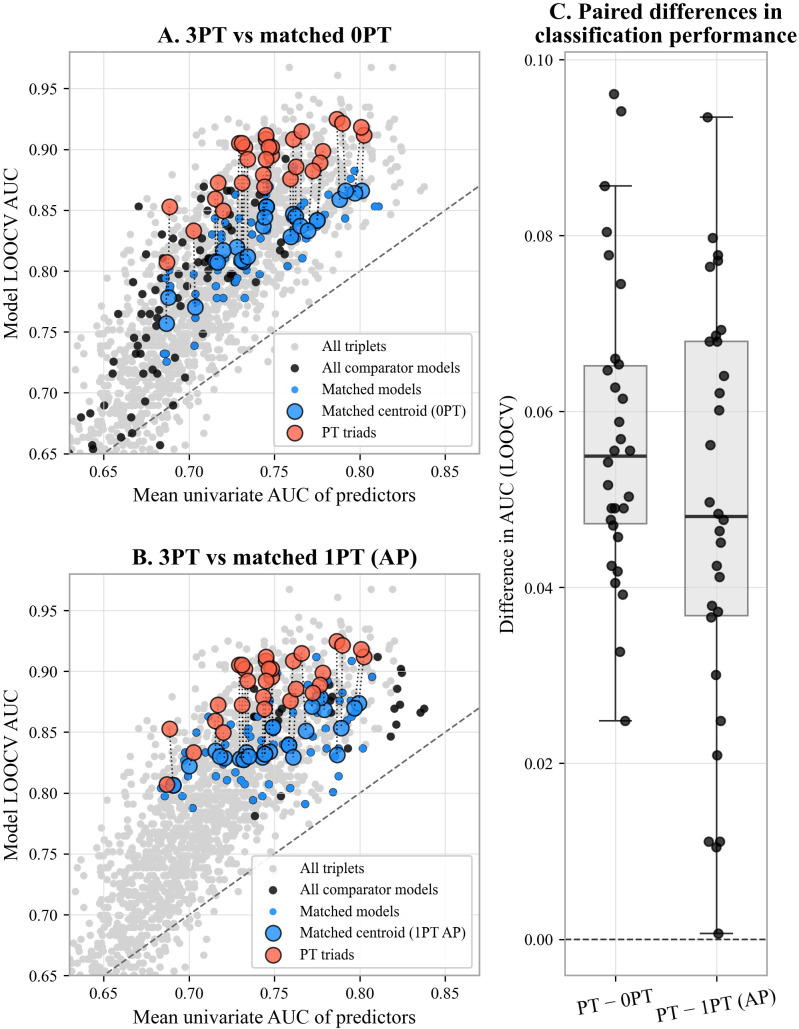
Paired comparisons of PT triplets vs matched non-PT triplets. **(A)** 3PT vs matched 0PT comparisons with matched centroids; **(B)** 3PT vs matched AP-only 1PT comparisons; **(C)** ΔAUC distributions for both contrasts. In **(A, B)**, each 3PT model is paired to a matched set of comparator models (matched on mean univariate AUC), summarized by the comparator-set centroid; connector segments link each 3PT model to its matched centroid, and the diagonal line indicates equality (ΔAUC = 0). 3PT/1PT/0PT, triplets containing 3/1/0 PT domains; ΔAUC, AUC_(3PT) − AUC_(matched centroid).

Using primary matching on mean univariate AUC with K = 5 nearest neighbors, PT triads showed consistently higher discrimination than matched 0PT triplets (median ΔAUC = 0.0549; central 95% of paired differences: 0.0307–0.0807; Wilcoxon *p* = 2×10^-6^) (**Figure 3**). This pattern persisted when comparing PT triads against matched AP-only 1PT triplets: median ΔAUC = 0.0480 (central 95% of paired differences: 0.0078–0.0758; Wilcoxon *p* = 2×10^-6^). This pattern suggests that PT-consistent structure carries complementary information beyond univariate trait strength alone.

Conclusions were unchanged under K = 1 and profile-matching variants (median ΔAUCs were 0.0507–0.0621; full results in **Supplementary Materials**).

### Can parsimonious, interpretable PT-models capture as much discriminative signal as high-dimensional benchmarks?

#### PT models vs high-dimensional benchmark

**Figure 4:**
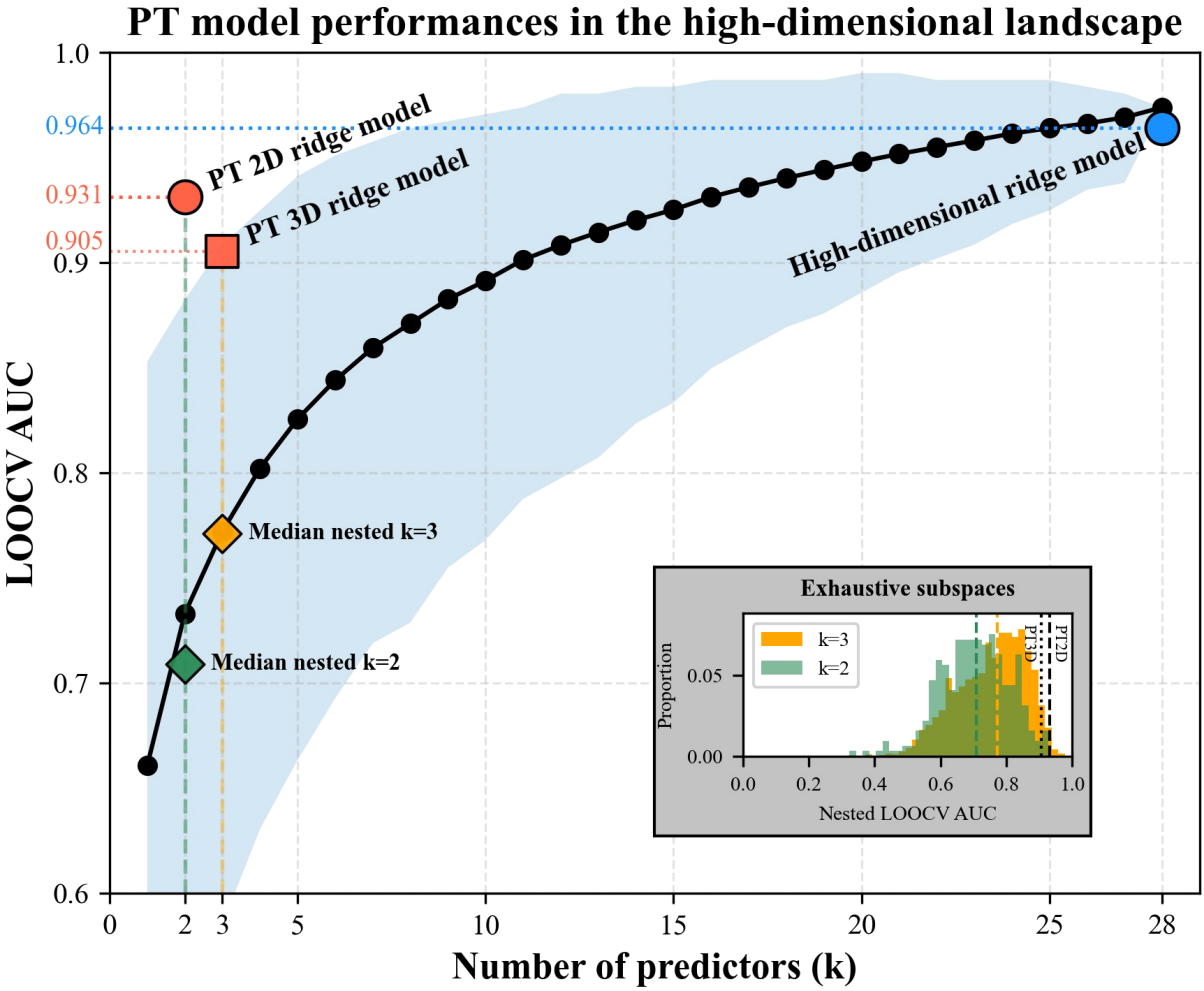
Model-size landscape of high-dimensional models with PT models overlaid. Main panel: AUC by model size k (median and 95% interval), with 2D/3D PT, 28D high-dimensional benchmark and k = 2–3 medians overlaid. Inset: AUC distributions for k = 2 and k = 3 models, highlighting PT model performances and subspace median performances (dashed lines). k, number of predictors; 2D/3D/28D, number of predictors/dimensions in the model. LOOCV, leave-one-out cross-validation; AUC, area under receiver operating characteristic; PT, pathogenetic triad.

Within the expanded predictor space, the 28D ridge model achieved AUC = 0.964 (95% CI 0.892–1.000), BA = 0.917 (0.824–1.000), and Brier = 0.088 (0.043–0.143). The 2D PT model achieved AUC = 0.931 (0.843–0.993), BA = 0.770 (0.628–0.900), and Brier = 0.124 (0.077–0.172). The 3D PT model achieved AUC = 0.905 (0.788–0.987), BA = 0.770 (0.618–0.893), and Brier = 0.137 (0.088–0.190). Thus, the parsimonious representation captured discrimination within approximately 0.03 AUC of the 28-variable benchmark in this dataset while remaining low-dimensional and interpretable (**Figure 4**).

#### Model-size randomization: where do the PT models sit?

Within the high-dimensional landscape of models of *k* = 1–28, median AUC increased steeply at small *k*, exceeding 0.90 by *k* = 11, and exceeding 0.95 by *k* = 20, with diminishing marginal gains thereafter (**Figure 4**). The PT 2D model (AUC = 0.931) intersected the landscape median near *k* ≈ 15, indicating that the 2D PT representation captured performance typical of substantially larger data-driven models in this predictor set.

In the *k* = 2 subspace, the PT 2D model ranked among the strongest *k* = 2 models, exceeding the 95^th^ percentile of the *k* = 2 distribution (p95 = 0.859; empirical one-sided *p <* 0.0031, defined as the proportion of *k* = 2 models exceeding the PT model AUC). In the *k* = 3 subspace, the PT 3D model achieved AUC = 0.905, ranking near the top of the *k* = 3 distribution, exceeding the 95^th^ percentile (p95 = 0.899; empirical one-sided *p* = 0.044, defined as the proportion of *k* = 3 models exceeding the PT model AUC). Together, these results indicate that PT-based parsimonious models are unusually strong within the low-dimensional spaces and can approximate the discrimination observed in substantially larger models in this dataset.

### Exploratory roadmap from multiverse mapping

Exploratory mapping identified multiple high-performing PT-consistent triads with strong univariate model AUC across pairings (**Supplementary Tables 5**, **6**, **Supplementary Figure 5**), indicating that performance is not driven by any single CC or NB operationalization.

## Discussion

This study provides preliminary evidence for the Pathogenetic Triad (PT) as a theory-guided multilevel framework for autism liability. In an IQ-matched case–comparison cohort, low-dimensional PT operationalizations showed high out-of-sample discrimination and outperformed strength-matched data-driven alternatives, while recovering much of the discrimination observed in higher-dimensional models. Despite the limited sample size, convergence across multiverse specifications suggests that the PT captures complementary configurational information in this pilot cohort.

### Configurational liability and theory-guided efficiency

The findings are consistent with the PT framework’s central claim that autism liability depends on the configuration of autistic traits (AP), cognitive capacity (CC), and neuropathological burden (NB), not trait severity alone (13, 14). While AQ was the strongest predictor, multiverse and matched-pair analyses showed that PT-consistent models outperformed AQ-only and non-triadic combinations matched on mean univariate input strenght, even when combinations included AQ. This pattern supports the interpretation that combining the three PT domains may integrate complementary information relative to other triplets with similar univariate strength in this dataset.

In line with this, the 2D PT model achieved strong cross-validated discrimination and calibration despite conservative IQ-matched conditions. Taken together–across the prespecified primary model, the multiverse results, and the matched comparisons–these patterns are consistent with the triad structure reflecting non-redundant structure in liability within this dataset, rather than merely bundling correlated predictors. Unsupervised clustering in PT space showed above-chance alignment with diagnosis, consistent with structure in the triad space that does not depend on supervised fitting.

Methodologically, this illustrates a simple point: theory can reduce overfitting by limiting what the model is allowed to consider. Standard regularization (e.g., ridge) controls model complexity *after* the feature set has been chosen, whereas the PT constrains the feature space *beforehand* by specifying a small set of mechanistic dimensions. In this sample, moving from the 2D to the 3D PT model slightly reduced performance. This should not be interpreted as evidence that CC and NB are empirically indistinguishable. Rather, in a small pilot cohort, it suggests that a theory-guided dimensional reduction in which the shared liability-relevant variance between CC and NB is captured efficiently by a composite axis, whereas modeling them separately introduces additional degrees of freedom that are more likely to reflect measurement-specific variation or noise.

Theory-guided models may therefore offer more interpretability and potentially improved generalizability in settings where multiple causal pathways converge on similar phenotypes. Single-level analyses (e.g., behavioral subdomain decomposition) (25) may miss interactions across levels that emerge only when traits, capacity, and burden are modeled jointly. Finally, a one-dimensional TriadIndex achieved comparable performance, supporting the feasibility of summarizing configurational liability into a practical additive risk-oriented score (discussed below).

### Parsimony and the cost of complexity

Most diagnostic signal in this dataset was recoverable with very low dimensionality. The PT models performed close to a 28-feature high-dimensional ridge model spanning additional behavioral and neurobiological domains (ΔAUC ≈ 0.03–0.05). In a small sample, the high-dimensional model is more sensitive to noise: added predictors yielded diminishing returns while increasing complexity and reducing interpretability.

The trade-off was visible in the model-size landscape. The 2D PT model performed best among all *k* = 2 models and matched the median performance trajectory at much larger *k* (≈ 15 predictors); the 3D PT model likewise ranked near the top of the *k* = 3 distribution. These patterns suggest that much of the predictive signal can be captured with only a few interpretable variables rather than distributing it across many loosely related features.

This parsimony is also practical: the PT axes correspond to interpretable constructs that can be visualized and linked to mechanistic hypotheses, whereas the 28-predictor model is too complex to interpret or communicate. In small clinical samples, a low-dimensional theory-guided representation can capture most discriminative signal while minimizing overfitting risk and maximizing transparency, offering a replication- and implementation-friendly starting point.

### The TriadIndex: quantifying configurational risk

We introduced the TriadIndex (TI) as a single, risk-oriented summary of an individual’s position in PT space. TI used an *a priori* 2:1:1 weighting, intended as a theory-guided starting point rather than a fitted optimum; in this sample it retained performance comparable to the multivariable PT model, consistent with an approximately one-dimensional liability axis. TI can be used as a pragmatic composite score, but it is best interpreted alongside its component values–similar to how total cholesterol is interpreted in conjunction with HDL and LDL. The current weighting is not proposed as universal, and replication studies should preregister a small group of plausible alternatives (e.g., up-weighting CC in cognitively unmatched samples). Notably, alternative simple weightings performed similarly, suggesting that including all PT components may matter more than fine-tuning their relative contributions.

### Measurement purity and construct validity

AP. AQ is practical and continuous but not domain-pure; some items may overlap with cognitive performance and internalizing symptoms, and autistic traits may fractionate into partially distinct subcomponents (e.g., social-communication vs. behavioral inflexibility) (26). Future work should therefore examine subscales, refined instruments, or latent AP factors integrating phenotypic, neurobiological, and genetic indicators to determine whether a cleaner AP operationalization sharpens the triad structure and reduces cross-loading with CC and NB-related constructs.

CC. WMIQ is a plausible proxy for compensatory capacity, but compensation is multi-faceted and unlikely to be exhausted by a single instrument. Broader executive, language-mediated, adaptive, and social-cognitive resources may also contribute to this domain. Future work should test whether a small composite provides a better operationalization of CC while preserving interpretability.

NB. HRV here functions as a proxy indicator for the NB domain in this operationalization. Resting vagal HRV (SD1) is mechanistically plausible but state-sensitive and only partially reflects burden-related developmental constraint; we do not assume that HRV is uniquely privileged as an NB indicator. Future work should compare repeated or context-varying autonomic indices, reactivity/recovery measures, and multimodal burden proxies, including neurobiological, genetic, and clinical markers. To probe this issue without treating individual downstream neurobiological end-result biomarkers as interchangeable with NB in the PT framework, we performed a supplementary restricted-domain comparison of triplets of the form AP + CC + X. In that analysis, models using NB as the third domain tended to occupy a higher-performing region than analogous substitutions from BEH or ANAT, while MEG substitutions were more competitive but not clearly superior (**Supplementary Figure 6**).

Future tests should directly compare genetically indexed PT components (e.g., polygenic trait and burden scores) and multimodal phenotypic proxies to evaluate domain fidelity. Improving measurement purity should reduce cross-domain collinearity, sharpen mechanistic interpretability, and provide a fairer test of whether the triad structure generalizes.

### Boundary conditions and generalizability

These findings should be interpreted as proof-of-concept under narrow boundary conditions. The balanced case–comparison design supports discrimination and calibration assessment (e.g., AUC/Brier) but does not support direct inference about screening utility in a real-world low-prevalence setting, where predictive values depend on base rates. The triad’s behavior may differ when components vary more widely, as in typical referral populations or mixed-sex and mixed-IQ samples.

Replication studies should broaden demographics (age, sex), increase heterogeneity along the CC-axis, and include differential diagnostic comparisons to evaluate specificity and transportability of the PT structure.

### Toward cumulative testing of PT

The exploratory analyses were used to support cumulative testing of the PT framework rather than to select a single “best” model. By evaluating multiple PT-consistent operationalizations we identified a set of models that performed well, listed in the Supplement, which should be prioritized for preregistered replication in new sampling contexts. By formally testing and presenting multiple PT models, one can aggregate results across studies and allow for structured meta-analyses to identify trends of performative models in relation to sample contingencies.

### Clinical interpretation: mechanistic profiling

Although this study does not support immediate clinical deployment of a predictive model, the PT framework may have conceptual utility as a mechanistic formulation lens. Rather than reducing assessment to diagnosis or a one-dimensional “severity”, the PT emphasizes the joint configuration of AP, CC, and NB. This configurational perspective may help explain variability in functional impact, timing of diagnosis, adaptive profile, and support needs across individuals who meet the same diagnostic criteria.

Under this framework, clinical interpretation shifts from asking “How autistic is this person?” to “What balance of liability, compensation, and burden is present?” For example, pronounced AP paired with strong compensatory capacity and low burden may be associated with later diagnosis and relatively preserved functioning, whereas a moderate AP combined with limited compensation and higher burden may correspond to earlier impairment or greater support needs despite lower trait severity. Such distinctions illustrate how similar observable behaviors may arise from different underlying configurations.

Importantly, these implications remain conceptual at this stage. If future preregistered external validation demonstrates transportability, calibration, and generalizability across diverse samples–including differential diagnostic comparisons–PT-derived summaries might inform structured case formulation by clarifying the interplay between liability, compensatory resources, and burden-related constraints. At present, however, the framework should be regarded as a theory-driven research model and interpretative aid rather than a deployable clinical test.

### Limitations

This study has several important limitations. First, the cohort was small and convenience-based (n = 42), increasing uncertainty around performance estimates and limiting the stability of model ranking. Although leakage-free nested cross-validation and permutation testing reduce optimistic bias, they do not substitute for independent external validation. Performance metrics in small samples may fluctuate substantially under resampling and should not be interpreted as stable population parameters.

Second, the sample was demographically restricted to adult males and employed IQ matching, which strengthens internal control but constrains variance along the CC-axis and limits immediate generalization to females, younger age groups, and cognitively heterogeneous clinical populations. The IQ-matched design also makes this a conservative test of CC-related contributions to liability.

Third, clinical diagnosis served as the case label, which is an imperfect proxy for underlying liability. Control status cannot exclude undiagnosed autism, and diagnostic misclassification would tend to attenuate separation. Future work should explicitly model sensitivity to plausible mislabeling rates.

Finally, all analyses were conducted in a single cohort. Despite strict leakage control, multiverse exploration, and permutation testing, the findings remain provisional until replicated across independent datasets, broader demographic strata, and differential diagnostic designs.

## Conclusions

This pilot study provides preliminary evidence for the Pathogenetic Triad (PT) as a theory-guided multilevel model for autism liability. Prespecified low-dimensional PT operationalizations showed high out-of-sample discrimination and higher discrimination than strength-matched, data-driven alternatives, consistent with configurational information beyond trait severity alone while capturing much of the discrimination broadly comparable to that observed in higher-dimensional models in this dataset.

External replication in larger, more diverse and differential-diagnostic samples is required, with preregistered operationalizations and prospective evaluation of calibration and generalizability. If replicated, the PT provides a falsifiable, mechanistically interpretable structure that can be re-operationalized across datasets and may support both interpretable stratification in research settings and mechanistic case formulation.

## Data Availability

The datasets presented in this article are not readily available because of ethical/consent constraints but are available from the corresponding author on reasonable request. To enable computational reproducibility, analysis scripts and a fully synthetic dataset are available at https://doi.org/10.17605/OSF.IO/SG8AD. Requests to access the datasets should be directed to DS, darko.sarovic@gu.se.
