# Supplementary material for: Preliminary testing of a prespecified liability architecture for autism: theory-guided pathogenetic triad models outperform strength-matched alternatives
Source: Front Psychiatry. 2026 Jul 6;17:1837909. doi: 10.3389/fpsyt.2026.1837909 (PMC13381483; doi:10.3389/fpsyt.2026.1837909)
Supplement: Supplementary file 1 [file DataSheet1.pdf]

Supplementary Methods and Results

The scripts and synthetic data can be found at:

https://doi.org/10.17605/OSF.IO/SG8AD

Table of Contents

S0. VARIABLES **2**

S1. ADDITIONAL PREPROCESSING DETAILS **3**

S1.1. UNIVARIATE CHARACTERIZATION AND HANDLING OF VARIABLE-SPECIFIC MISSINGNESS 3
S1.2. ECG PREPROCESSING AND HRV METRICS 7

S2. NESTED CROSS-VALIDATION PIPELINE **7**

S3. UNSUPERVISED K-MEANS CLUSTERING IN TRIAD SPACE **9**

S4. TRIADINDEX (TI) IMPLEMENTATION DETAILS AND WEIGHTING SENSITIVITY **9**

S5. TRIPLET MULTIVERSE OF LOGISTIC MODELS **12**

S5.1. MULTIVERSE ENUMERATION AND LOGISTIC REGRESSION 12
S5.2. PT TRIADS IN THE K=3 DOMAIN-RESTRICTED MULTIVERSE 13
S5.3. THIRD-DOMAIN SUBSTITUTION ANALYSIS: AP + CC + X 15

S6. MATCHING SENSITIVITY ANALYSES FOR PT VS COMPARATOR TRIPLETS **16**

S7. CANDIDATES FOR PREREGISTRATION **19**

S7.1. PT CANDIDATES 20
S7.2. TI CANDIDATES (AP:CC:NB WEIGHTING) 21

S8. SYNTHETIC DATASET GENERATION AND DISCLOSURE CHECKS **21**

S9. REFERENCES **21**

S0. Variables

Variable names are presented below. In the rest of the supplement, any prefix denotes the domain that variable belongs to (e.g., AP_AQ).

AQ = Autism Spectrum Questionnaire

WMIQ = Working Memory Intelligence Quotient (IQ)

SIQ = Speed IQ

PIQ = Performance IQ

FSIQ = Full-Scale IQ

VIQ = Verbal IQ

CVI = Cardiac Vagal Index

SD1 = Standard Deviation 1 of the Pointcaré plot

perc_RR50 = percent of inter-beat-intervals differing by more than 50ms

SD2 = Standard Deviation 2 of the Pointcaré plot

CSI = Cardiac Sympathetic Index

SDNN = Standard Deviation of successive inter-beat-intervals

SPSensSeek = Sensory Profile (SP) Sensation Seeking

SPLowReg = SP Low Registration

TAS = Toronto Alexithymia Scale

SPSensAvoi = SP Sensory Avoidance

SPSensSens = SP Sensation Sensitivity

SPtotal = SP total score

hippV = Hippocampal volume (sum of left and right)

totalSV = Total Subcortical gray matter Volume

amygV = Amygdala volume (sum of left and right)

totalGV = Total Gray matter Volume

totalWV = Total White matter Volume

M170amp = Magnetoencephalographic (MEG) 170ms amplitude

lateamp = MEG late component amplitude

M130lat = MEG 130ms latency

M170lat = MEG 170ms latency

M130amp = MEG 130ms amplitude

S1. Additional preprocessing details

The main manuscript summarizes preprocessing at a high level. Here we provide the operational rules used in the scripts for normality screening, log transformation, and univariate characterization, because these steps define the predictor distributions used by all downstream models.

Normality screening was performed separately for each candidate predictor using the Shapiro–Wilk test and the distributional moment of skewness on the raw scale (Supplementary Table 1). A predictor was flagged as a log-transform candidate if it showed evidence of non-normality (Shapiro–Wilk $p < 0.05$) and meaningful positive skew (skew > 0.5).

For candidates, a 'safe log' transform was applied: if the minimum value was > 0, we used log(x). If any values were ≤ 0, we shifted the variable by $|\min(x)| + 1e{-}6$ before logging, i.e., $\log(x + |\min(x)| + 1e{-}6)$. This ensures the transform is defined while preserving rank ordering.

In the present dataset, four variables met the screening criteria and were log-transformed: NB_CSI, NB_SDNN, NB_perc_RR50, and MEG_M170amp (Supplementary Table 1).

Supplementary Table 1. Normality screening results for variables flagged for log transformation (raw scale). All flagged variables were log-transformed using the 'safe log' rule described in S1.

Variable	n	Shapiro-W	p	Skew	Kurtosis
NB_CSI	42	0.855969	8.82633e-05	1.69088	3.66029
NB_SDNN	42	0.814907	9.35117e-06	1.63954	2.48858
MEG_M170amp	39	0.90467	0.0030111	1.33076	2.66696
NB_perc_RR50	42	0.868997	0.000192369	1.11707	0.432446

S1.1. Univariate characterization and handling of variable-specific missingness

Univariate summaries were computed for each predictor using all available participants for that variable (pairwise availability), so sample sizes differ across predictors. Supplementary Table 2 lists per-group subsample sizes, means and standard deviations, effect sizes (Hedges' g; autism minus non-autism), and univariate in-sample AUCs (visualized in Supplementary Figure 1).

Univariate AUC_raw was almost perfectly correlated with absolute standardized mean differences (|Hedges' g|; $r = 0.982$), indicating that using AUC as the primary effect-size display does not materially alter predictor ranking relative to standardized mean differences.

For multiverse comparisons and matching, univariate AUCs were expressed as discriminative strength (AUC), defined as max(AUC_raw, 1 − AUC_raw), so that all values lie in [0.5, 1.0] regardless of direction of effect.

Supplementary Table 3 presents cross-validated univariate AUC's, Brier and BA for all variables, using the same leakage-controlled evaluation pipeline as the primary model.

Supplementary Table 2. Univariate descriptive statistics and discriminative strength (area under curve, AUC) for all candidate predictors. AUC = max(AUC_raw, 1−AUC_raw).

Variable	Domain	n	n_autism	Autism (mean ± standard deviation)	Non-autism (mean ± standard deviation)	Hedges' g (A-C)	AUC (in-sample)
AQ	AP	42	21	24.57 ± 8.86	11.90 ± 6.02	1.641	0.882
WMIQ	CC	42	21	103.29 ± 13.90	114.00 ± 12.35	-0.800	0.721
SIQ	CC	42	21	96.19 ± 18.92	107.48 ± 13.23	-0.678	0.709
PIQ	CC	42	21	116.95 ± 20.32	111.90 ± 13.57	0.287	0.617
FSIQ	CC	42	21	109.62 ± 15.47	114.33 ± 11.37	-0.341	0.598
VIQ	CC	42	21	113.71 ± 17.61	116.62 ± 12.53	-0.186	0.563
CVI	NB	42	21	4.56 ± 0.40	4.90 ± 0.21	-1.020	0.786
SD1	NB	42	21	124.30 ± 63.05	185.05 ± 53.94	-1.016	0.785
perc_RR50	NB	42	21	1.44 ± 1.37	2.66 ± 0.92	-1.027	0.764
SD2	NB	42	21	357.79 ± 115.59	450.88 ± 81.93	-0.912	0.728
CSI	NB	42	21	1.14 ± 0.28	0.92 ± 0.20	0.860	0.727
SDNN	NB	42	21	4.58 ± 0.68	4.50 ± 0.50	0.133	0.542
SPSensSeek	BEH	40	19	40.53 ± 6.40	48.57 ± 6.06	-1.267	0.816
SPLowReg	BEH	40	19	33.05 ± 7.98	26.43 ± 7.00	0.868	0.763
TAS	BEH	42	21	48.71 ± 10.85	39.67 ± 9.47	0.872	0.724
SPSensAvoi	BEH	41	20	37.80 ± 7.47	32.00 ± 7.13	0.779	0.687
SPSensSens	BEH	41	20	36.30 ± 9.55	31.10 ± 7.35	0.601	0.657
SPtotal	BEH	40	19	147.58 ± 21.78	138.10 ± 19.50	0.451	0.635
hippV	ANAT	40	20	8753.22 ± 976.28	7261.41 ± 1116.06	1.395	0.848

totalSV	ANAT	40	20	64620.30 ± 4792.76	58158.65 ± 5915.05	1.176	0.823
amygV	ANAT	40	20	3489.72 ± 391.87	3070.43 ± 557.92	0.852	0.742
totalGV	ANAT	40	20	703918.22 ± 52352.74	662122.16 ± 57444.86	0.745	0.720
totalWV	ANAT	40	20	500020.42 ± 52101.41	459566.62 ± 63308.88	0.684	0.720
M170amp	MEG	39	20	1.84 ± 0.39	2.31 ± 0.47	-1.059	0.787
lateamp	MEG	39	20	0.39 ± 2.06	2.03 ± 1.84	-0.825	0.703
M130lat	MEG	39	20	0.11 ± 0.02	0.10 ± 0.02	0.440	0.642
M170lat	MEG	39	20	0.17 ± 0.02	0.16 ± 0.02	0.359	0.596
M130amp	MEG	39	20	-5.75 ± 4.15	-5.58 ± 4.23	-0.039	0.526

Supplementary Figure 1. Univariate diagnostic resolution of candidate predictors and group distributions of core PT components.

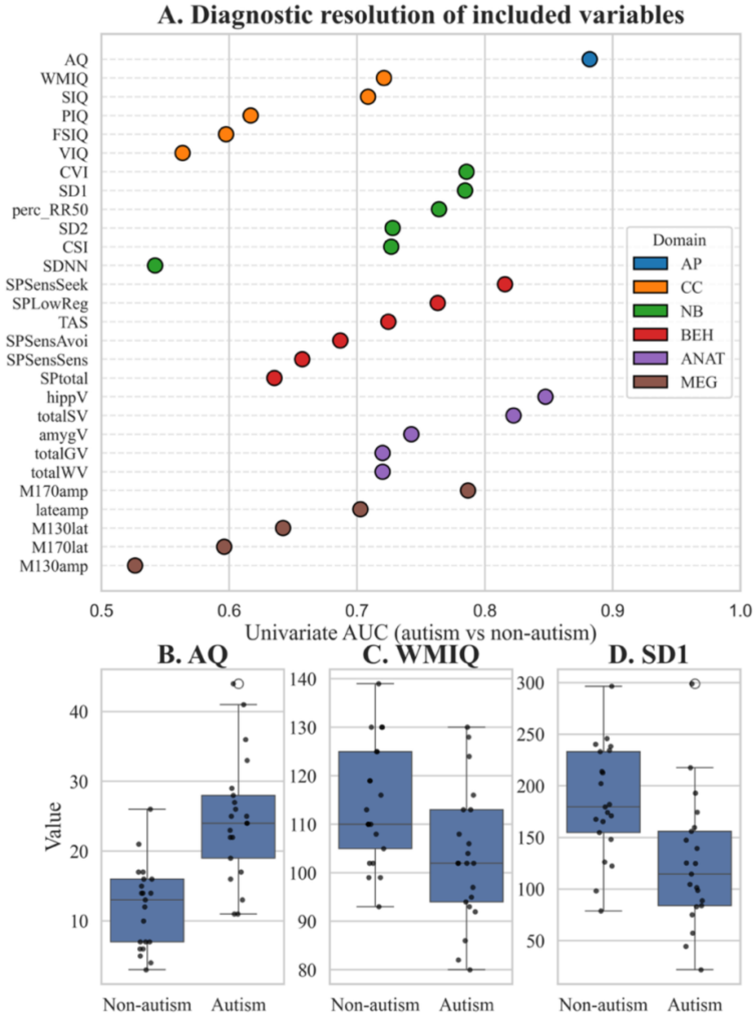

(A) Univariate AUC for each predictor (autism vs non-autism), sorted by domain. (B-D) Group distributions for AQ, WMIQ, and SD1, with group contrasts: AQ p =

3×10^{-6}, Hedges' g = *+1.64; WMIQ* p = *0.01,* g = *−0.80; SD1* p = *0.002,* g = *−1.02 (*g *computed as autism–non-autism). (Abbrev: AQ = Autism-Spectrum Quotient; WMIQ = Working Memory Index (WAIS); SD1 = short-term HRV (Poincaré SD1).)*

Supplementary Table 3. Out-of-sample single-predictor performance using nested leave-one-out cross-validation.

Variable	Domain	AUC	Brier	Balanced Accuracy
AQ	AP	0.862	0.163	0.833
WMIQ	CC	0.660	0.250	0.738
SIQ	CC	0.664	0.245	0.667
PIQ	CC	0.574	0.250	0.643
FSIQ	CC	0.565	0.253	0.643
VIQ	CC	0.463	0.253	0.571
CVI	NB	0.741	0.222	0.714
SD1	NB	0.714	0.240	0.738
perc_RR50	NB	0.696	0.232	0.738
SD2	NB	0.667	0.236	0.667
CSI	NB	0.671	0.246	0.714
SDNN	NB	0.422	0.250	0.595
SPSensSeek	BEH	0.799	0.205	0.752
SPLowReg	BEH	0.704	0.241	0.752
TAS	BEH	0.673	0.227	0.667
SPSensAvoi	BEH	0.629	0.239	0.631
SPSensSens	BEH	0.624	0.249	0.681
SPtotal	BEH	0.539	0.254	0.644
hippV	ANAT	0.820	0.196	0.825
totalSV	ANAT	0.750	0.231	0.750
amygV	ANAT	0.670	0.247	0.725
totalGV	ANAT	0.710	0.246	0.750
totalWV	ANAT	0.762	0.253	0.775
M170amp	MEG	0.797	0.211	0.792
lateamp	MEG	0.600	0.251	0.643
M130lat	MEG	0.553	0.252	0.639

M170lat	MEG	0.563	0.252	0.618
M130amp	MEG	0.092	0.250	0.500

AUC, Brier score, and balanced accuracy were calculated from out-of-fold predictions. Each variable was evaluated separately as a single-predictor ridge-logistic model using the same leakage-free nested LOOCV framework as the primary classifier.

S1.2. ECG preprocessing and HRV metrics

A single-channel ECG was recorded concurrently with MEG. R-peaks were detected using MNE-Python's ECG event detection (find_ecg_events with a set ECG channel and event_id; QRS threshold set to auto). For high-sampling-rate recordings (5 kHz), the ECG was resampled to 1 kHz and band-pass filtered (1–35 Hz, FIR) prior to peak detection to improve robustness. Interbeat intervals (IBIs; ms) were computed as differences between consecutive R-peak times.

Artifact handling. HRV was computed from IBIs after conservative, rule-based screening aimed at excluding physiologically implausible or clearly artifactual intervals. Specifically, IBIs were flagged if they fell outside broad physiologic bounds (290–2200 ms) and if they showed abrupt, implausible beat-to-beat changes (a maximum-change rule). Additional outlier screens based on distributional criteria for IBIs and successive differences were applied. Heuristic screening was used for conservative artifact flagging with visual QC. The final HRV calculations used the remaining IBIs intervals.

Metric estimation. Time-domain HRV metrics were computed using standard definitions ("Heart rate variability. Standards of measurement, physiological interpretation, and clinical use. Task Force of the European Society of Cardiology and the North American Society of Pacing and Electrophysiology," 1996).

S2. Nested cross-validation pipeline

All supervised models were evaluated with leakage-free nested cross-validation unless otherwise stated. The outer loop used leave-one-out cross-validation (LOOCV), holding out one participant at a time as the test case. All data-dependent steps–including scaling, construction of composite variables, and hyperparameter selection–were performed using only the training participants within each outer split, and the learned transformations were then applied to the held-out participant.

Model and determinism. Ridge-penalized logistic regression was implemented with an L2 penalty using the liblinear solver (max_iter = 10,000; class_weight = None). Random seeds were fixed to ensure determinism (global seed = 12345). Thread counts are not hard-capped in the scripts; users can enforce single-thread determinism via environment variables if needed.

Inner-loop tuning of C. Within each outer training set, the regularization parameter C was tuned using inner stratified K-fold cross-validation (default K = 5, shuffle = True)

optimizing log loss. If class counts in the training set were insufficient to support the requested number of splits, K was reduced automatically to the largest feasible value (minimum 2) based on per-class counts in that outer training fold. The candidate grid for C comprised 17 log-spaced values from 10^{-4} to 10^{4}. Model selection used the 1-SE rule: we identified the C with the lowest mean log loss across inner folds, computed its standard error, and selected the most regularized model (smallest C) whose mean log loss was within one standard error of the minimum. The kitchen-sink landscape used a computationally simplified evaluation with fixed C; as described under "Kitchen-sink landscape" in the main Methods.

Out-of-fold predictions and metrics. Discrimination was summarized primarily using AUC computed from out-of-fold decision scores. To make AUC comparable across outer folds and models, AUC was computed from intercept-free decision scores (i.e., the linear predictor without the intercept term, xβ), which removes fold-specific intercept shifts. Thresholded performance was summarized using balanced accuracy (BA). The classification threshold was selected within each outer training set by maximizing BA on cross-validated training predictions (generated without using the held-out participant) and then applied to the held-out participant. Calibration was summarized using the Brier score computed from out-of-fold predicted probabilities and visualized using four-bin calibration plots based on out-of-fold probabilities.

Uncertainty intervals. Confidence intervals reported were obtained by nonparametric bootstrap resampling of participants (2,000 resamples). Each resample drew participants with replacement and recomputed the metric from the paired out-of-fold predictions and observed labels; the 2.5th and 97.5th percentiles of the bootstrap distribution were taken as the 95% interval.

Permutation test (primary model). The primary model used label-permutation testing implemented by shuffling diagnostic labels and rerunning the full LOOCV pipeline for each permutation (N_perm = 20,000 by default), yielding a null distribution of LOOCV AUCs. The empirical p-value was computed as (count(AUC_perm ≥ AUC_obs) + 1) / (N_perm + 1). The results are presented in Supplementary Figure 2.

Supplementary Figure 2. Permutation-based null distributions of PT model performance.

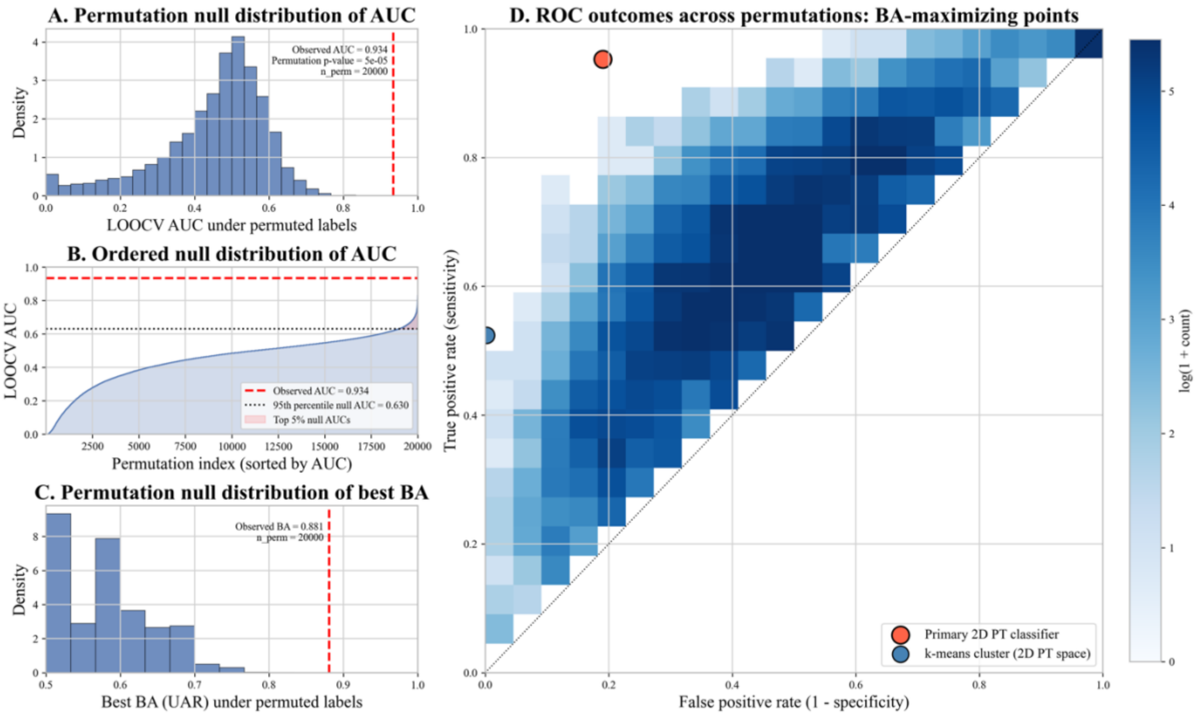

(A) Distribution of AUCs under label permutations; red line indicates observed AUC. (B) Ordered AUCs from all permutations, with the observed AUC and the null distribution 95th percentile. (C) Distribution of the best balanced accuracy (BA) under label permutations, red line indicates observed BA. (D) Heatmap of permutation operating points corresponding to BA, highlighting the PT model (red) and the k-means clustering solution (blue).

Missingness control (complete-case subsets). Unless otherwise specified, model comparisons within a given model family were conducted on complete-case subsets defined for that family to ensure that differences in missingness did not vary across models within the same comparison.

S3. Unsupervised k-means clustering in triad space

Clustering was performed on triad space data for z(AP) and z(NB/CC) using scikit-learn's KMeans with n_clusters=2 and n_init=50 random initializations, retaining the solution with the lowest within-cluster sum of squares. Since k-means labels are arbitrary, we aligned cluster labels to the diagnostic groups by choosing the labelling (0 vs 1) that maximized balanced accuracy. Sensitivity, specificity, and BA were then computed by comparing the aligned cluster labels with autism vs non-autism status. The operating point is shown in Supplementary Figure 2.

S4. TriadIndex (TI) implementation details and weighting sensitivity

The TriadIndex (TI) collapses triad space to a one-dimensional score by taking a weighted sum of standardized triad components. Standardization of each component

was performed within each LOOCV training fold and then applied to the held-out participant. Cognitive capacity and vagal HRV were treated as protective (lower values imply higher risk), so their standardized values were sign-inverted before combining them.

Calibration plots for TI used four fixed probability bins with edges [0, 0.25, 0.5, 0.75, 1.0].

Two TI weighting schemes were evaluated (Supplementary Table 4): a prespecified scheme (2:1:1; AP:CC:NB) and an equal-weights scheme (1:1:1). Supplementary Table 4 shows the results of each model and Supplementary Figures 3 and 4 reproduce the TI-by-diagnosis plot, ROC curve, and calibration summary used for the primary model in the main manuscript, for the prespecified and equal weights schemes respectively.

Supplementary Table 4. TriadIndex weighting sensitivity. All metrics are LOOCV; 95% intervals are bootstrap intervals across participants (2,000 resamples).

TI model	w_AP	w_CC	w_NB	C used for TI model	LOOCV AUC	AUC 95% CI low	AUC 95% CI high	LOOCV Brier	Brier 95% CI low	Brier 95% CI high	LOOCV BA	BA 95% CI low	BA 95% CI high
TI specified (2:1:1)	0.500	0.250	0.250	3.162	0.943	0.857	1.000	0.108	0.063	0.163	0.905	0.844	1.000
TI equal (1:1:1)	0.333	0.333	0.333	1.000	0.948	0.872	0.995	0.120	0.078	0.165	0.881	0.825	0.979

Supplementary Figure 3. TriadIndex (TI) as a one-dimensional PT representation using a 2:1:1 weighting (AP:CC:NB).

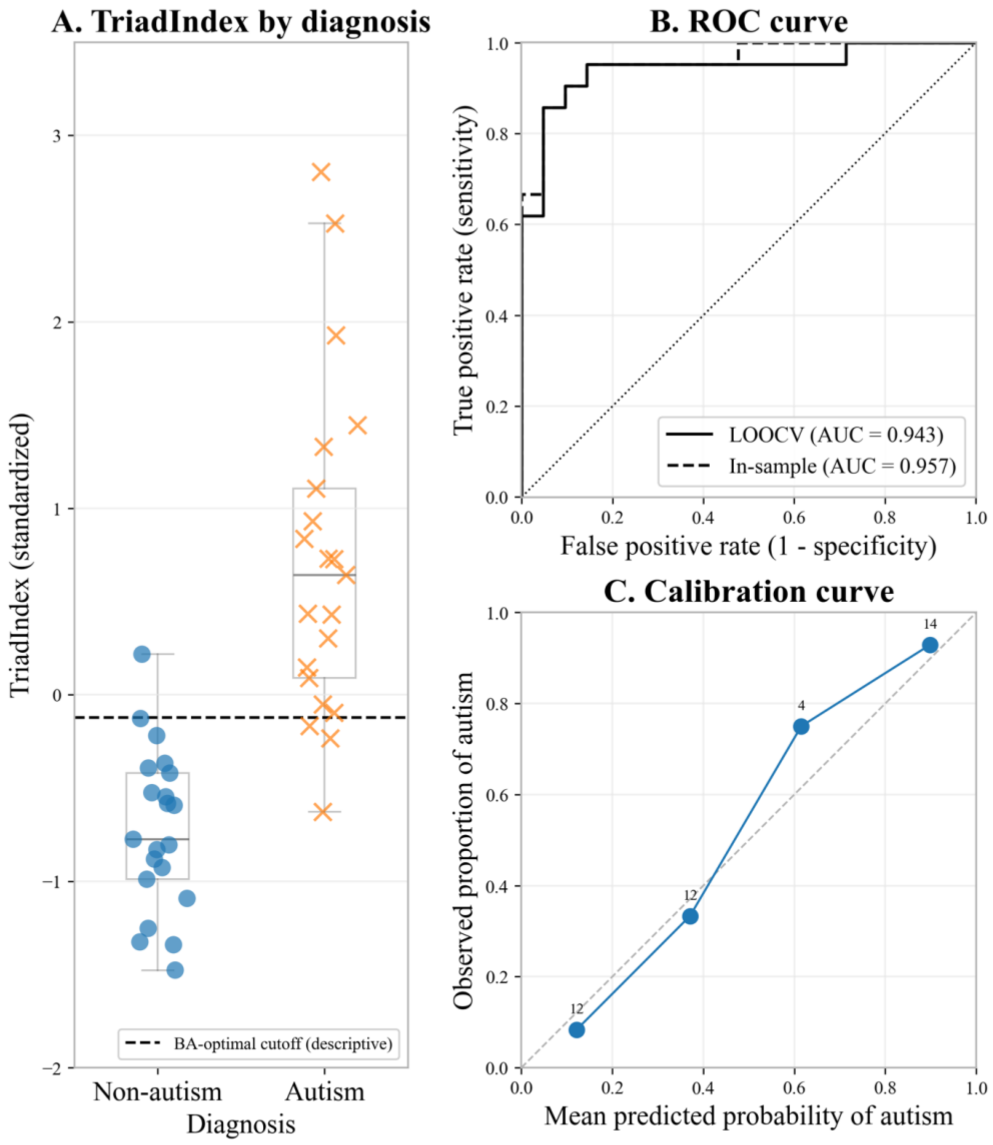

(A) TI by diagnosis. (B) ROC curves using in-sample (dashed line) and cross-validation decision scores (solid line). (C) Calibration plot (number indicates bin size).

Supplementary Figure 4. TriadIndex sensitivity analysis using equal weights (1:1:1 for AP:CC:NB).

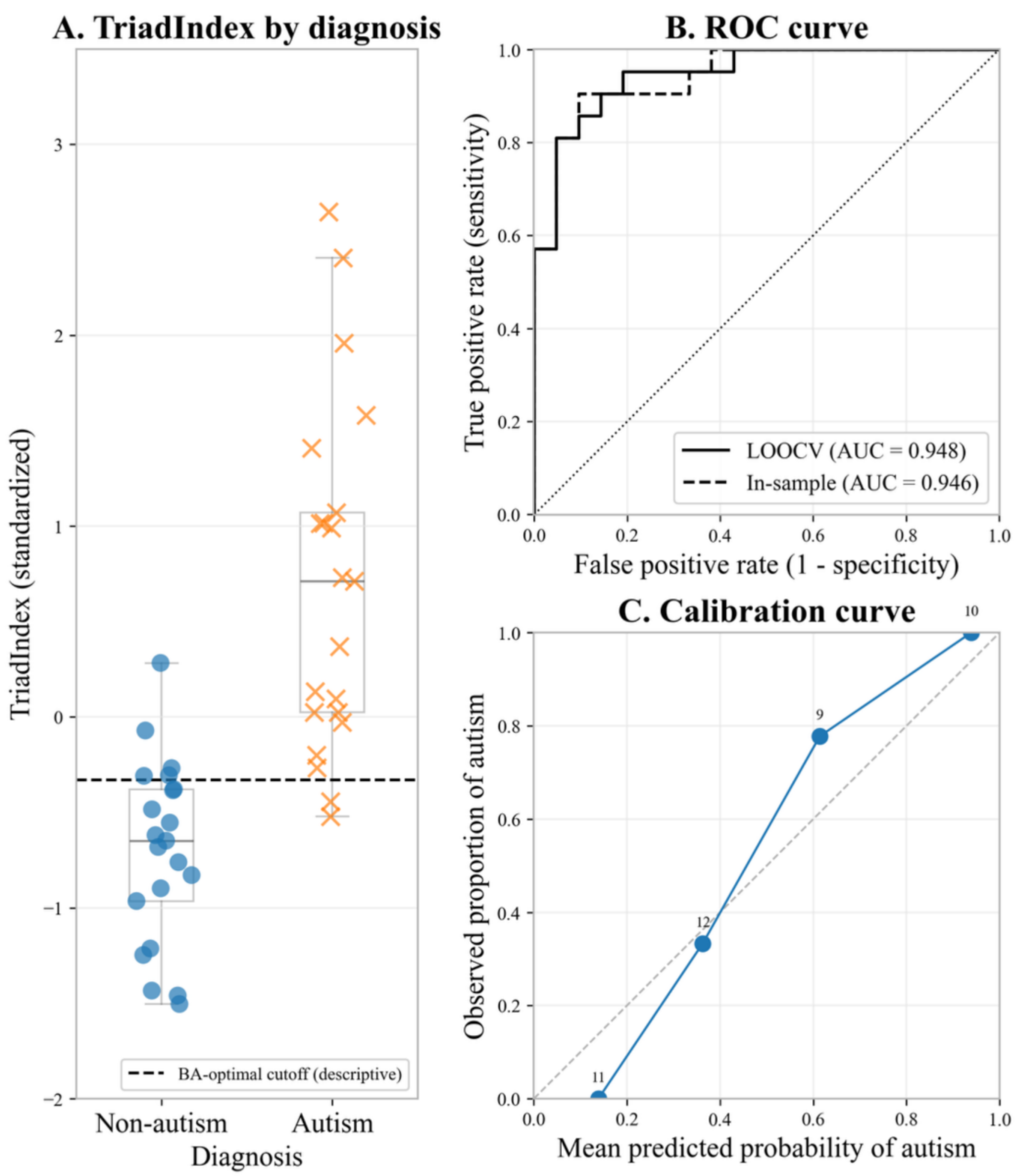

(A) TI by diagnosis, (B) ROC curve (LOOCV and in-sample), and (C) calibration plot using four probability bins with bin counts shown.

S5. Triplet multiverse of logistic models

S5.1. Multiverse enumeration and logistic regression

We enumerated all triplets, subject to the constraint that each model contained at most one variable per domain (AP, CC, NB, BEH, ANAT, MEG). Across the dataset, we used the complete-case subset, so that all triplets across the multiverse would have equal sample size (n = 35). For each candidate combination we implemented the full nested cross-validation pipeline described in the Analytic overview of the main manuscript, fitting penalized logistic regression models with scikit-learn's LogisticRegression with penalty="l2" (ridge), solver="liblinear", max_iter=10000,

with random_seed fixed for reproducibility, and obtained fold-centered probabilities which were used to calculate AUC. For each model we recorded: model size; predictor identities; domain counts per model (n_AP, n_CC, n_NB, n_BEH, n_ANAT, n_MEG); number of PT domains (n_PT = n_AP + n_CC + n_NB); sample size used (*n*); LOOCV AUC; and mean univariate AUC (average of univariate AUC of the model's predictors).

S5.2. PT triads in the k=3 domain-restricted multiverse

From this model space, we identified the 30 PT-consistent triads (one AP, one CC, and one NB predictor from the PT candidate set).

Supplementary Table 5 lists the PT-consistent triads and their performances. Across the 30 PT triads, LOOCV AUC ranged from 0.807 to 0.925 (median 0.894). The top-performing PT triads primarily combined AQ and WMIQ with alternative NB proxies (e.g., SD2, CVI, and %RR50).

Supplementary Table 5. PT-consistent triads (n=30) from the k=3 domain-restricted multiverse (fixed complete-case subset, n=35). Mean univariate AUC is the mean discriminative strength of the three predictors in the triad, computed on the same fixed subset.

AP predictor	CC predictor	NB predictor	Mean univariate AUC	LOOCV AUC	LOOCV Brier	n	Median C (inner CV)
AP_AQ	CC_WMIQ	NB_SD2	0.786	0.925	0.127	35	3.162
AP_AQ	CC_WMIQ	NB_perc_RR50	0.790	0.922	0.142	35	3.162
AP_AQ	CC_WMIQ	NB_SD1	0.801	0.918	0.131	35	1.000
AP_AQ	CC_SIQ	NB_perc_RR50	0.766	0.915	0.127	35	3.162
AP_AQ	CC_PIQ	NB_CVI	0.745	0.912	0.132	35	3.162
AP_AQ	CC_WMIQ	NB_CVI	0.802	0.912	0.138	35	3.162
AP_AQ	CC_FSIQ	NB_CVI	0.761	0.908	0.139	35	3.162
AP_AQ	CC_FSIQ	NB_SD2	0.745	0.908	0.149	35	3.162
AP_AQ	CC_PIQ	NB_SD2	0.729	0.905	0.134	35	3.162
AP_AQ	CC_VIQ	NB_SD2	0.731	0.905	0.149	35	3.162
AP_AQ	CC_PIQ	NB_perc_RR50	0.733	0.902	0.147	35	3.162
AP_AQ	CC_SIQ	NB_CSI	0.748	0.902	0.137	35	1.000
AP_AQ	CC_VIQ	NB_CVI	0.747	0.902	0.144	35	3.162
AP_AQ	CC_SIQ	NB_CVI	0.778	0.899	0.132	35	3.162
AP_AQ	CC_FSIQ	NB_perc_RR50	0.748	0.895	0.148	35	3.162

AP_AQ	CC_VIQ	NB_SD1	0.745	0.892	0.144	35	1.000
AP_AQ	CC_VIQ	NB_perc_RR50	0.734	0.892	0.150	35	3.162
AP_AQ	CC_SIQ	NB_SD1	0.777	0.889	0.133	35	1.000
AP_AQ	CC_SIQ	NB_SD2	0.763	0.886	0.139	35	3.162
AP_AQ	CC_WMIQ	NB_CSI	0.772	0.882	0.144	35	1.000
AP_AQ	CC_PIQ	NB_SD1	0.743	0.879	0.151	35	3.162
AP_AQ	CC_FSIQ	NB_SD1	0.759	0.876	0.146	35	1.000
AP_AQ	CC_FSIQ	NB_CSI	0.731	0.873	0.157	35	1.000
AP_AQ	CC_VIQ	NB_CSI	0.717	0.873	0.161	35	1.000
AP_AQ	CC_WMIQ	NB_SDNN	0.744	0.869	0.142	35	1.000
AP_AQ	CC_PIQ	NB_CSI	0.715	0.859	0.165	35	1.000
AP_AQ	CC_VIQ	NB_SDNN	0.688	0.853	0.170	35	1.000
AP_AQ	CC_SIQ	NB_SDNN	0.720	0.850	0.158	35	1.000
AP_AQ	CC_FSIQ	NB_SDNN	0.703	0.833	0.168	35	1.000
AP_AQ	CC_PIQ	NB_SDNN	0.687	0.807	0.190	35	1.000

To summarize which predictors tended to appear in stronger PT triads, we computed, for each predictor, the mean LOOCV AUC across all PT triads containing that predictor (Supplementary Table 6; Supplementary Figure 5). This descriptive summary is intended to guide preregistered follow-up hypotheses rather than to select an optimal feature set. Values differ from univariate AUC due to complementarities within triads. The summary suggested that AQ, several IQ indices (particularly WMIQ and SIQ), and the HRV indices CVI, SD2 and %RR50 tended to appear in higher-performing PT models, whereas SDNN tended to appear in comparatively weaker PT models.

Supplementary Table 6. Predictor-wise mean LOOCV AUC across PT-consistent triads containing that predictor (n=30 PT triads).

**Predictor (variable)**	**Mean LOOCV AUC across PT triads**	**Rank (1=best)**
NB_CVI	0.907	1
NB_SD2	0.906	2
NB_perc_RR50	0.905	3
CC_WMIQ	0.905	4
NB_SD1	0.891	5
CC_SIQ	0.890	6
AP_AQ	0.888	7

CC_VIQ	0.886	8
CC_FSIQ	0.882	9
NB_CSI	0.878	10
CC_PIQ	0.877	11
NB_SDNN	0.842	12

Supplementary Figure 5. Predictor-wise mean LOOCV AUC across PT triads.

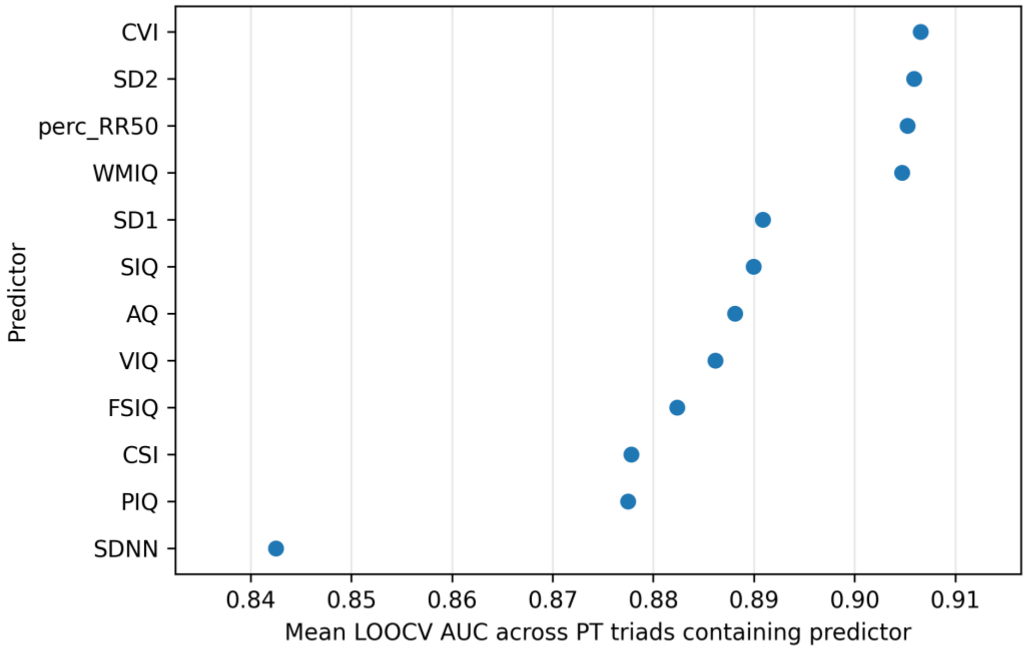

S5.3. Third-domain substitution analysis: AP + CC + X

Because NB was the least direct and empirically established component of the present PT operationalization, we performed an additional exploratory analysis focused on the third domain paired with AP and CC. We restricted the k = 3 multiverse to models containing exactly one AP predictor, one CC predictor, and one additional predictor from NB, BEH, ANAT, or MEG. This fixed comparison asks whether the HRV-derived NB domain behaves favorably relative to available non-PT third-domain alternatives when the AP+CC structure is held constant.

As shown in Supplementary Figure 6, AP+CC+NB models occupied the upper portion of this restricted model space. Behavioral and anatomical substitutions generally showed lower LOOCV AUCs, suggesting that adding another autism-relevant behavioral or structural measure to AP and CC did not reproduce the same performance pattern. MEG substitutions were more competitive and in some cases reached the performance frontier despite lower mean univariate input strength, indicating that functional neurophysiological measures may contain complementary information relevant to autism classification. This analysis does not establish convergent validity of HRV as a direct measure of NB, but it shows that the prespecified HRV-derived NB

operationalization was not inferior to available alternative third-domain substitutions in the present sample and identifies MEG as a candidate domain for future multimodal NB-related operationalizations.

Supplementary Figure 6. Third-domain substitution among AP+CC triplets.

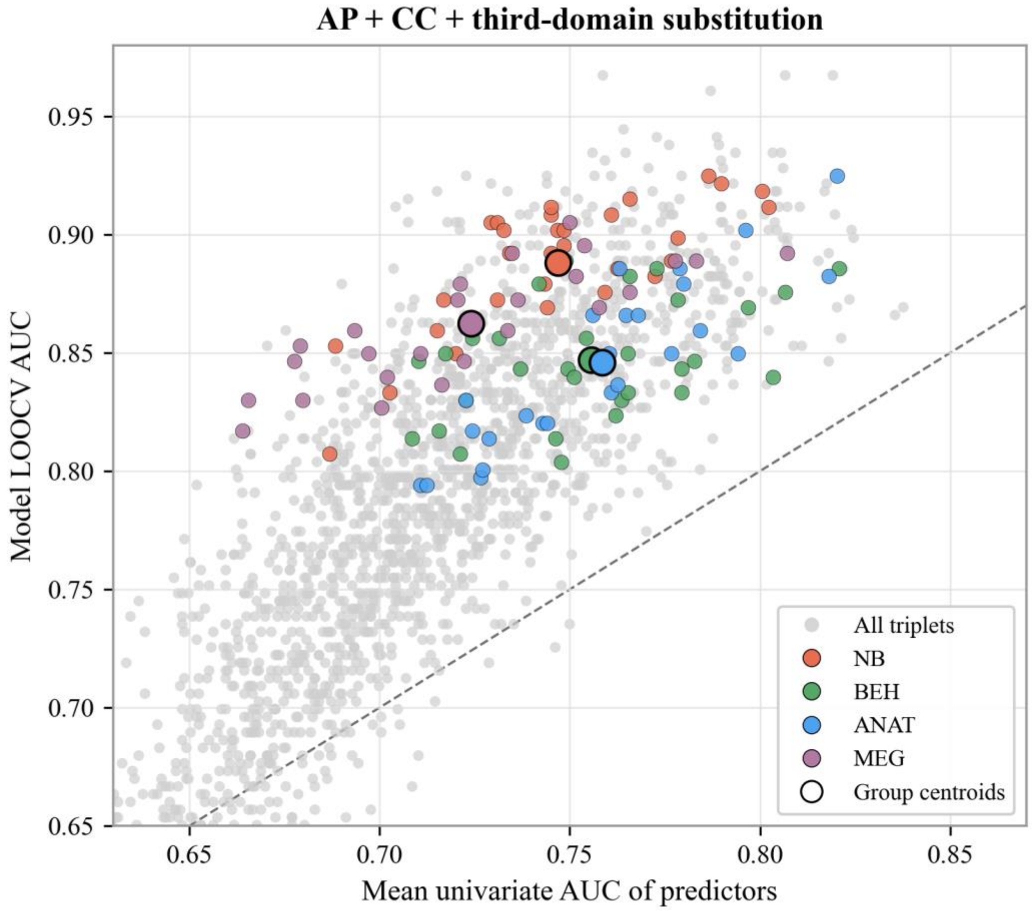

Each colored point represents a domain-restricted k = 3 model containing exactly one Autistic Personality (AP) predictor, one Cognitive Capacity (CC) predictor, and one additional predictor from either Neuropathological Burden (NB), Behavioral (BEH), Anatomical (ANAT), or Magnetoencephalography (MEG) domains. Grey points show all domain-restricted k = 3 triplets from the full multiverse for context. The x-axis shows mean univariate area under the receiver operating characteristic curve (AUC) across the three predictors, used as an estimate of input strength, and the y-axis shows nested leave-one-out cross-validated (LOOCV) AUC. Larger outlined points indicate group centroids.

S6. Matching sensitivity analyses for PT vs comparator triplets

The main manuscript compares PT triads (3PT) against two comparator sets: triplets containing no PT domains (0PT) and triplets containing only an AP-domain predictor (1PT(AP-only)). Comparator triplets were selected by matching on univariate signal to reduce the risk that PT triads appear superior simply because they contain stronger individual predictors.

The primary matching scheme used k-nearest-neighbor matching (K = 5) on mean univariate AUC of the three predictors (XONLY matching). Two sensitivity analyses were conducted and are reported here: (1) single-neighbor matching (K = 1) under the same XONLY criterion (Supplementary Figure 7), and (2) matching on the full 3D univariate-AUC profile (one value per predictor in the triplet) using K = 5 neighbors (Supplementary Figure 8).

For each PT triad, we computed the paired difference in LOOCV AUC between the PT triad and each of its matched comparator triplets, then summarized these paired differences across PT triads. Statistical testing used a Wilcoxon signed-rank test across PT triads, and uncertainty in the mean paired difference was summarized with a bootstrap interval across PT triads (2,000 resamples). Supplementary Table 7 provides the full summary across matching schemes.

Supplementary Table 7. Matched-pair sensitivity analyses for PT vs comparator triplets.

Matching scheme	Comparison	Mean ΔAUC (PT − comparator)	Median ΔAUC	P25	P75	95% CI low (mean)	95% CI high (mean)	Wilcoxon p	Unique matched models	Reuse factor
PRIMARY_XONLY_k5	PT triads vs 0PT	0.057	0.055	0.047	0.065	0.051	0.064	2×10^{-6}	63	2.381
PRIMARY_XONLY_k5	PT triads vs 1PT(AP-only)	0.049	0.048	0.037	0.068	0.040	0.057	2×10^{-6}	62	2.419
SENS_XONLY_k1	PT triads vs 0PT	0.054	0.051	0.033	0.072	0.045	0.063	2×10^{-6}	22	1.364
SENS_XONLY_k1	PT triads vs 1PT(AP-only)	0.063	0.062	0.042	0.087	0.052	0.073	2×10^{-6}	20	1.500
SENS_3DPROFILE_k5	PT triads vs 0PT	0.061	0.057	0.044	0.082	0.053	0.069	2×10^{-6}	43	3.488
SENS_3DPROFILE_k5	PT triads vs 1PT(AP-only)	0.049	0.051	0.031	0.074	0.039	0.058	3×10^{-6}	50	3.000

ΔAUC is defined as LOOCV AUC(PT triad) − LOOCV AUC(comparator). 'Reuse factor' quantifies how often the same comparator model is reused across PT triads.

Supplementary Figure 7. Sensitivity analysis using single-neighbor matching (XONLY, K=1).

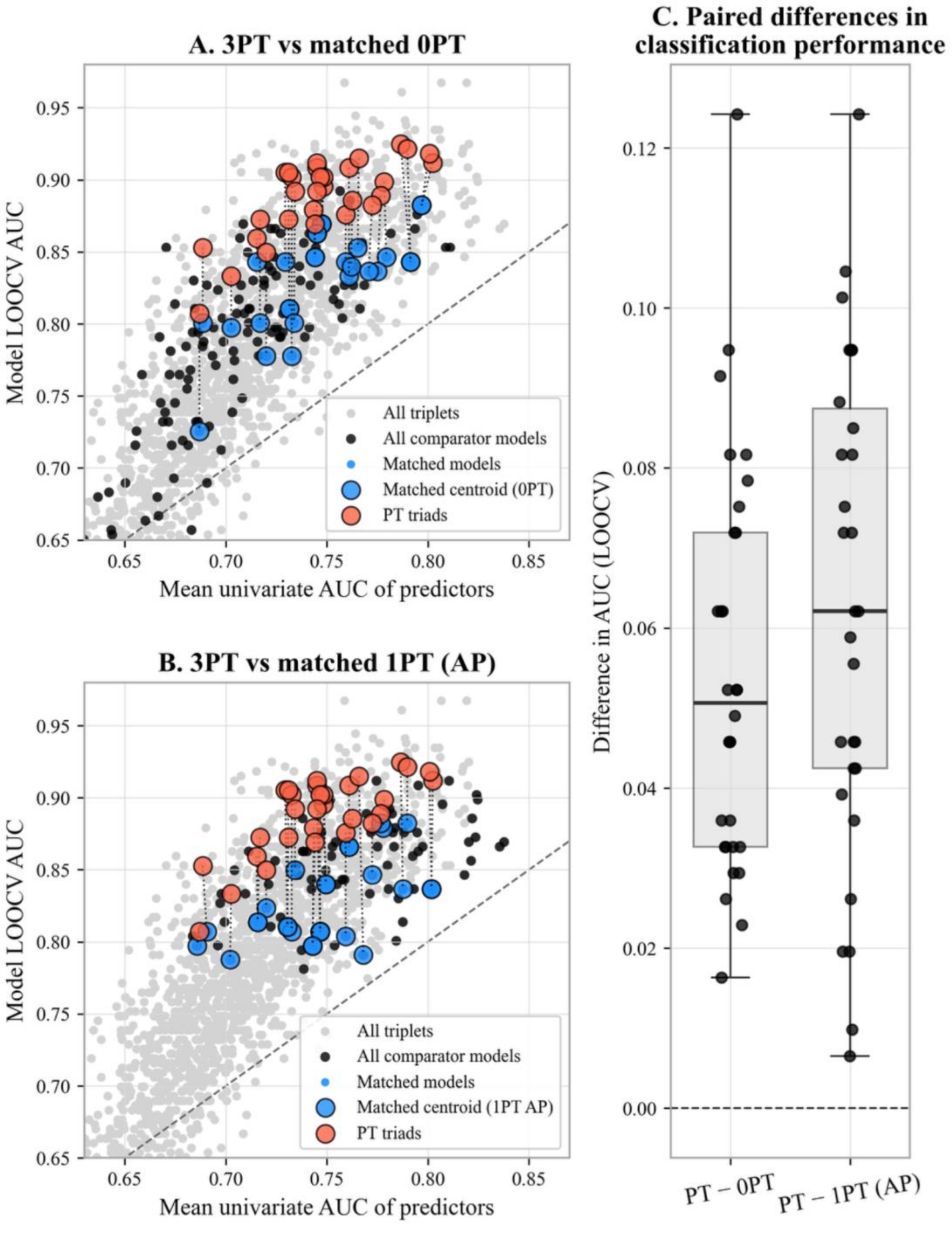

Panels match the main figure: PT triads (red) compared with matched 0PT and 1PT(AP-only) comparator triplets (blue) within the full triplet universe (gray).

Supplementary Figure 8. Sensitivity analysis using 3D-profile matching (K=5).

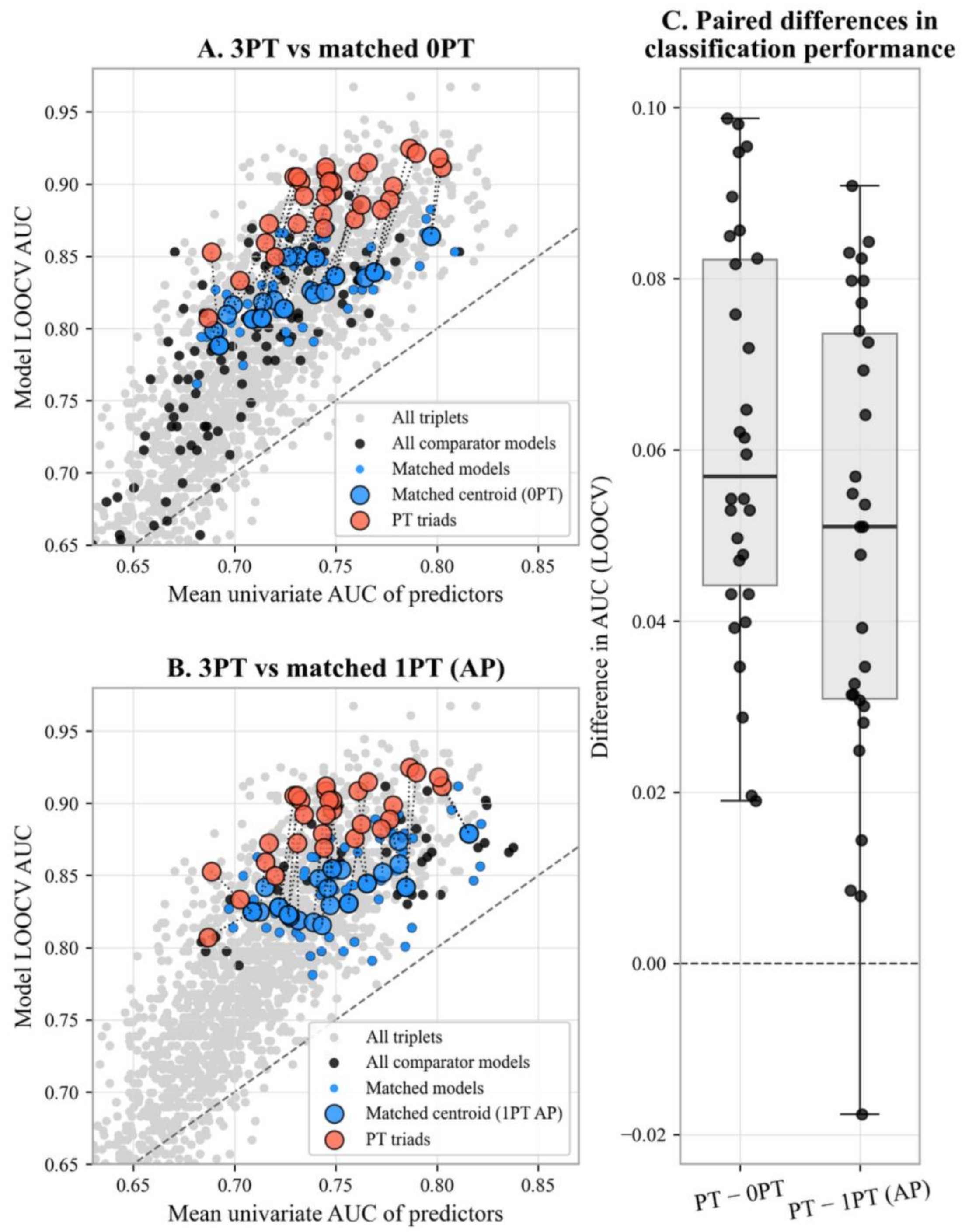

Comparator triplets are matched to each PT triad using the three univariate AUC values (one per predictor) rather than the mean alone.

S7. Candidates for preregistration

Since this dataset was recorded in a small, demographically homogeneous cohort during specific conditions, transportability of suggested candidates is limited both by limited generalizability (due to sample idiosyncrasies) and changing contingencies (different recording circumstances, most notably for HRV metrics, here sampled during

a simple cognitive task during MEG scanning). With this limitation in mind, one can still specify candidates to be tested in addition to the primary model and TI-model.

These candidates can be tested with Holm correction. For example, the primary model, and the three best performing PT models in the k=3 space can be tested with alpha-thresholds of 0.05, 0.025, 0.0125, and 0.00625, such that if any model fails its alpha, the rest of the models are not tested.

In external validation, candidate models are evaluated using both discrimination and calibration, with uncertainty quantified using confidence intervals. Discrimination is assessed primarily by AUC (with bootstrapped 95% confidence intervals), and replication is interpreted as supportive if performance is broadly consistent with the present study while allowing for case-mix differences; practically, one can require that replication AUC remains in an acceptable range (e.g., AUC ≥ 0.80 and/or a lower 95% CI bound ≥ 0.70) and that any decline relative to the present estimate is not large (e.g., ΔAUC ≤ 0.05–0.10). Calibration is assessed using intercept (calibration-in-the-large) and slope, supported by calibration plots and the Brier score. Replication calibration is considered acceptable if the intercept is near 0 and the slope is near 1 (e.g., slope 0.8–1.2, with confidence intervals including 1), indicating limited systematic over- or underprediction and limited overfitting. Brier score is reported alongside the null-model Brier (predicting the observed prevalence only), and improvement over the null is considered supportive. Validation targets should be prespecified and applied consistently across candidate models.

S7.1. PT candidates

The following PT-based model are prioritized for cumulative testing in external validation. For all models, predictors are standardized within the training data and applied to the held-out participant(s) using the same leakage-free pipeline as in the main analyses.

The core confirmatory model with AQ, WMIQ, SD1 as a 2D model:

1) z(AQ) and mean(z(WMIQ), z(SD1))

Sensitivity variants for the primary 2D model with weighted mean for z-scores within the NB/CC composite, to test relative contributions of NB and CC in 2D triad space (similar to the approach for the TI model weighting):

2) z(AQ) and weighted mean(2 * z(WMIQ), 1 * z(SD1))
3) z(AQ) and weighted mean(1 * z(WMIQ), 2 * z(SD1))

The top three performing PT triplets as 2D models:

4) z(AQ) and mean(z(WMIQ), z(SD2)): highest LOOCV AUC in this sample
5) z(AQ) and mean(z(WMIQ), z(%RR50)): taps into a distinct HRV property
   z(AQ) and mean(z(WMIQ), z(SD1)): the primary model

By combining the two highest ranking predictors each from CC and NB domains (Supplementary Table 6) we can construct an additional four candidate triplets (three after deduplication):

6) z(AQ) and mean(z(WMIQ), z(CVI))
   z(AQ) and mean(z(WMIQ), z(SD2))
7) z(AQ) and mean(z(SIQ), z(CVI))
8) z(AQ) and mean(z(SIQ), z(SD2))

S7.2. TI candidates (AP:CC:NB weighting)

One can also evaluate a small set of prespecified TI-weightings applied to fold-standardized domain scores. Supplementary Table 8 lists potential models for preregistration and their performances in this sample.

Supplementary Table 8. TI-weightings and model performances

Rationale	Weighting (AP:CC:NB)	LOOCV AUC (in this sample)
Theory-informed, AP-dominant	2:1:1	0.943
AP-dominant, with stronger CC contribution	4:2:1	0.914
AP-dominant, with stronger NB contribution	4:1:2	0.952
Theory-agnostic equal weights	1:1:1	0.948
Exploratory, pilot-optimized	7:1:3	0.959

S8. Synthetic dataset generation and disclosure checks

Because participant-level data cannot be shared, we provide a fully synthetic dataset for pipeline execution and code inspection. Synthetic records were generated within each diagnostic group using a Gaussian-copula approach: numeric variables were rank-transformed to normal scores, a dependence structure was estimated in that space, multivariate samples were drawn, and values were mapped back to the original scales via inverse empirical cumulative distribution function (quantile) mapping. Categorical variables were sampled from within-group empirical frequencies. Missingness was applied post hoc to approximate observed missingness rates. The synthetic dataset is intended for computational reproducibility and is not expected to reproduce reported effect sizes or predictive model performance.

To reduce disclosure risk, we ran automated sanity checks comparing real and synthetic data: (i) exact row-match screening (overall and by group; expected 0), (ii) nearest-neighbor distances between datasets in standardized feature space (synthetic→real and real→synthetic; avoiding very small distances), and (iii) a density-robust ratio comparing each real participant's nearest-synthetic distance to their nearest-real-neighbor distance (ratios substantially <1 would be undesirable).

S9. References

Heart rate variability. Standards of measurement, physiological interpretation, and clinical use. Task Force of the European Society of Cardiology and the North

American Society of Pacing and Electrophysiology. (1996). *Eur Heart J*, *17*(3), 354-381. https://www.ncbi.nlm.nih.gov/pubmed/8737210
